# Projecting the potential distribution of *Rickettsia japonica* in China and Asian adjacent regions under climate change using the Maxent model

**DOI:** 10.3389/fpubh.2025.1478736

**Published:** 2025-03-06

**Authors:** Xiaoxu Wang, Meng Shang, Zihao Wang, Haoqiang Ji, Zhenxu Wang, Qiyong Liu

**Affiliations:** ^1^Department of Vector Control, School of Public Health, Cheeloo College of Medicine, Shandong University, Jinan, China; ^2^National Key Laboratory of Intelligent Tracking and Forecasting for Infectious Disease, Department of Vector Biology and Control, National Institute for Communicable Disease Control and Prevention, Chinese Center for Disease Control and Prevention, Beijing, China; ^3^School of Public Health, Nanjing Medical University, Nanjing, China

**Keywords:** *Rickettsia japonica*, Japanese spotted fever, potential distribution, Maxent, climate change

## Abstract

**Objective:**

To determine the current and future suitable areas of *Rickettsia japonica*, and to provide a reference for preventing its outbreak and spread.

**Methods:**

Based on the geographic distribution of *R. japonica* and *Haemaphysalis longicornis* overlapping data points and information on 56 climatic factors, we utilized the Maxent model to estimate suitable areas for *R. japonica* in Asian adjacent Regions and China. Model parameter adjustments and the construction of receiver operating characteristic curves were conducted using R 4.3.0 software.

**Results:**

Average precipitation in June (prec6, 28.2%), Temperature Seasonality (bio4, 9.8%) and the minimum temperature in August (tmin8, 9.2%) contributed most to the distribution of *R. japonica*. The performance metrics for the Maxent model in predicting the distribution of *R. japonica* are as follows: the Area Under the Curve (AUC) is 0.990, the True Skill Statistic (TSS) is 0.857, and the Kappa statistic is 0.763. Under current climatic conditions, the Asian and adjacent space medium and highly suitable areas for *R. japonica* are estimated to be 176.78 × 10^4^ km^2^ and 95.13 × 10^4^ km^2^, respectively. The highly suitable areas for *R. japonica* were mainly distributed in east and south Asia. In China, the high suitability areas are mainly distributed in the southeast coastal areas and the Qinling Mountains and Huai River cities. Under future climatic conditions, the Asian and adjacent regions maximum area change rate of *R. japonica* increased by 118.65%, and that of China increased by 50.42%. Meanwhile, the suitable areas of *R. japonica* gradually expanding northward in China.

**Conclusion:**

Under global climate change, the suitable area of *R. japonica* is generally increasing, with a northward shift observed in China. Governments should strengthen monitoring, risk assessment, and response strategies in highly suitable regions, while also preventing the invasion of *R. japonica* from external source.

## 1 Introduction

Ticks, belonging to the Ixodoidea family, stand out as critical vectors for diseases affecting both humans and animals, surpassing other invertebrate carriers in the diversity of pathogens they transmitted. Tick-borne rickettsioses represent a globally distributed zoonotic threat caused by the obligate intracellular bacteria, rickettsia (1). Japanese spotted fever (JSF) emerges as a severe acute tick-borne disease, attributed to an alpha-proteobacterial agent (*Rickettsia japonica*) belonging to the spotted fever group within the genus Rickettsia (2). The presence of *R. japonica* has been documented in various regions, including Japan, South Korea, and Thailand, with reports dating back to 1984 (3). Subsequent findings have identified the bacterium in both ixodes ticks and the blood of individuals in central, southeastern, and northeastern China (4–6). A large number of studies have shown that *Haemaphysalis longicornis* is the main vector for *R. japonica* (5, 7, 8). The clinical manifestation of JSF is characterized by a sudden onset of symptoms, including headache, fever, shaking chills, skin eruptions, tick bite eschars, and overall malaise (9). Research indicates that following infection with JSF, if not promptly treated, there is a possibility of developing eruptive purpura, disseminated intravascular coagulation complications, and multiple organ failure (10, 11). Hence, it is crucial to conduct public health surveillance to detect cases and outbreaks promptly, enabling the implementation of preventive measures. This underscores the significance of identifying potential high-risk areas (12).

Global climate change is reshaping the distribution and density of tick populations by altering annual mean temperature, annual precipitation, and temperature seasonality, leading to extreme dry months (13). Climate significantly influences species distribution by modifying life cycles and environmental variables such as temperature, precipitation, and wind speed. These changes can directly impact ecology, resulting in population increases or decreases, and potentially leading to species evolution or extinction (14). Consequently, climate change poses an urgent and potentially irreversible threat to human societies, fauna, and vectors. According to the IPCC report, human activities have caused global warming of approximately 1°C above preindustrial levels, with a probable range of 0.8°C to 1.2°C. It is expected that global warming will reach 1.5°C between 2030 and 2052 if it continues at the current rate (15). Numerous studies and models (16–18) predict a significant expansion in tick habitats due to climate change. The northern global climate is anticipated to become warmer and wetter, facilitating the survival and adaptation of ticks like *Haemaphysalis longicornis (H. longicornis)* in many regions (19). This expansion of ticks has been observed on a broad scale across regions like Japan and the wider Asian continent (20). Therefore, the growing need to predict the impact of global changes has driven ecology towards becoming a more predictive science (21).

Even though the precise worldwide distribution of JSF remains unclear, ecological niche models have established associations between species occurrence and diverse environmental and climatic factors. These models enable the anticipation of distribution maps (22). GARP, Bioclim, Domain, and Maxent are extensively utilized for predicting species distribution, with the Maximum Entropy model (Maxent) proving particularly effective on a global scale (23, 24). Maxent asserts that, given precisely stated prior data, the probability distribution that most accurately reflects the current knowledge state of the system is the one with maximum entropy. Lorenz et al. employed Maxent modeling to identify regions with the highest prevalence of Mayaro virus occurrence in South America. Their study, based on confirmed cases, serological detection spanning the last two decades, and socio-environmental variables, aimed to map out areas of heightened risk for Mayaro virus presence in the region (12). Cheng et al. utilized epidemiological models and Maxent to assess the potential risk map of the spread of Usutu virus in Europe, providing data for the epidemiology and risk assessment of disease transmission (25).

There are few studies on risk prediction of *R. japonica* and JSF, and the classification of risk areas is crucial for formulating public policy related to control and prevention. Given this context, we used overlapping datasets on the global distribution of *R. japonica* potential vectors ticks and *R. japonica* to identify potential areas where JSF occurs on a global scale and assess the influential bioclimatic variables for JSF using Maxent. Furthermore, it also aims to identify prospective areas for conservation planning under future climate change scenarios.

## 2 Materials and methods

### 2.1 Data collection

Distribution data on *R. japonica* and *H. longicornis* were obtained by retrieving data and information from the Global Biodiversity Information Facility (GBIF) (https://www.gbif.org, accessed on 1 March 2024) and the literature database (CNKI, PubMed, ScienceDirect, and Web of Science) (26). Both literature searches used combinations of the following search terms: “*R. japonica*” OR “*H. longicornis*” OR “Japanese spotted fever” to search in the database. Records lacking geographic coordinates were georeferenced in Google Maps (http://www.google.cn/intl/zh-CN/earth/, accessed on 5 March 2024) (27). Further cross-validation was conducted using Amap, and the results were verified and corrected through expert review. At a 95% confidence interval, the error margin is approximately 10 meters. Initially, 60 distribution coordinates of *R. japonica* and 203 distribution coordinates of *H. longicornis* were collected. To ensure data integrity, we meticulously scrutinized and removed duplicates and missing records, resulting in 52 and 139 valid distribution points for *R. japonica* and *H. longicornis*, respectively. We employed the INSIDE operation within ArcGIS software (version 10.7, ESRI Inc., United States) to exclude distribution points situated in marine environments. Subsequently, to mitigate spatial sampling bias and reduce model overfitting, we utilized ENMTools to filter the occurrence data, retaining only one point per grid cell at a spatial resolution of 5 arcminutes (28). Following these procedures, we obtained 48 reliable overlapping data distributions for *H. longicornis* and *R. japonica* (see Supplementary Figure 1 and coordinate point data). Given that the distribution of data points is primarily concentrated in Asia, we will delineate the geographical scope to Asia and adjacent regions to ensure the reliability of the model’s predictive extrapolation. To align with the specifications of the Maxent software (version 3.4.4, http://biodiversityinformatics.amnh.org/open_source/maxent; accessed date: 15 September 2023), the distribution records were formatted as csv files.

### 2.2 Climatic variables and processing

Climatic data were obtained from the WorldClim Global Climate Database (version 2.1, https://worldclim.org/; accessed on: 6 September 2023) (29). The dataset included near-current climate information spanning from 1970 to 2000, as well as projections for future climate conditions covering the periods 2021–2040, 2041–2060, 2061–2080, and 2081–2,100. These projections were based on the BCC-CSM2-MR model, a middle-resolution climate system model developed by Beijing and participating in CMIP6, considering four socio-economic pathways (SSPs): 126, 245, 370, and 585. Key climatic variables examined in this study comprised elevation (Ele), monthly minimum temperature (Tmin1–Tmin12), monthly maximum temperature (Tmax1–Tmax12), monthly precipitation (Prec1–Prec12), and nineteen bioclimatic variables (bio1–bio19; refer to Supplementary Table 1) at a spatial resolution of 5 arcminutes (approximately 10 km). To meet the requirements of the Maxent software, all climatic variables were converted into ASCII format (30).

To avoid overfitting due to high correlation among climatic variables, a preliminary experiment was conducted using the Maxent model with the distribution data of *R. japonica* and 19 environmental variables to obtain the contribution rates of climatic variables affecting *R. japonica* and rank them from high to low, subsequently discarding factors with a contribution rate less than 1.0%. The climatic variables initially selected were subjected to Pearson correlation analysis using R software (version 4.3.0 https://www.r-project.org; Obtained on April 15, 2022), and variables with a correlation coefficient absolute value greater than 0.8 and lower contribution rates were removed. Ultimately, 8 variables were chosen as predictors (Table 1).

**Table 1:** Percentage contributions of environmental variables in Maxent for *R. japonica.*

Abbreviation	Definition	Contribution rate (%)
prec6	Average Precipitation of June (mm)	28.2
bio4	Temperature Seasonality (standard deviation ×100)	9.8
tmin8	Minimum temperature of August (°C)	9.2
ele	Elevation	8.4
prec9	Average Precipitation of September (mm)	3.1
bio14	Precipitation of Driest Month	2.5
prec8	Average Precipitation of August (mm)	1.2
prec11	Average Precipitation of November (mm)	1.1

### 2.3 Optimizing parameters for the maximum entropy model

The Maxent software employs the maximum entropy method to model species niches and distributions. In this study, we aimed to forecast suitable areas for *R. japonica* under both near-term and future climate scenarios. The data sets were randomly split, allocating 75% of the distribution points for model establishment and 25% for testing. Bootstrap sampling was utilized, with the process involving 20 replicates, a limit of 5,000 iterations, and the application of a rule for the 10th percentile training presence threshold. In addition, 10-fold cross-validation was applied to the training and validation process of the model.

The optimization process of Maxent model parameters involved evaluating the influence of regularization multiplier (RM) and feature combination (FC) on predictive performance and accuracy (31, 32). RM was examined across eight levels: 0.5, 1, 1.5, 2, 2.5, 3, 3.5, and 4, while FC consisted of five characteristic parameters: automatic linear (L), quadratic (Q), fragmentation (hinge, H), product (P), and threshold (T), resulting in eight feature combinations (L, LQ, LQP, QHP, LQH, LQHP, QHPT, and LQHPT). The optimization of these parameters utilized the “ENMeval” package in the R software (33, 34), utilizing the Akaike Information Criterion Correction (AICc). Generally, smaller AIC values were prioritized in simulations, with AICc serving as a standard measure for assessing model fit quality (35). In this study, linear, quadratic, and product features were selected, with a regularization multiplier set at 1 (Parameter selection in Supplementary materials). The predictive performances and contributions of all the selected variables were determined using the jackknife test.

### 2.4 Model evaluation

For the evaluation of the Maxent model in this study, we employed three standard metrics: the Receiver Operating Characteristic (ROC) curve, the KAPPA statistic, and the True Skill Statistic (TSS). The ROC metric is determined by computing the area under the curve (AUC). The area measure has a value range of 0–1, with a value closer to 1 indicating a stronger correlation between environmental variables and the predicted geographic distribution of species, reflecting a higher predictive performance of the model (36). The KAPPA statistic is a normalized measure used to evaluate the agreement between model predictions and observed data, incorporating aspects of species range, sensitivity (True Positive Rate, TPR), and specificity (1 – False Positive Rate, FPR). The TSS provides a numerical index that represents the net accuracy of the model in predicting the presence and absence of species. The criteria for assessing the effectiveness and accuracy of these three-evaluation metrics are presented in Table 2 (37, 38).

**Table 2:** Model evaluation index reference standard.

Evaluation index	Failure	General	Moderate	Better	Excellent
ROC	0.00–0.60	0.60–0.70	0.70–0.80	0.80–0.90	0.90–1.00
TSS	0.00–0.40	0.40–0.55	0.55–0.70	0.70–0.85	0.85–1.00
KAPPA	0.00–0.20	0.20–0.40	0.40–0.60	0.60–0.80	0.80–1.00

### 2.5 Classification of suitable areas

The State Key Laboratory for Infectious Disease Prevention and Control of the Chinese Center for Disease Control and Prevention obtained the ArcGIS software. The probability of *R. japonica* presence was stratified into four categories using natural break points: unsuitable, low suitability, moderate suitability, and high suitability areas. This classification was performed using the reclassification tool in ArcGIS, based on complementary clog log values (39).

## 3 Results

**Figure 1:**
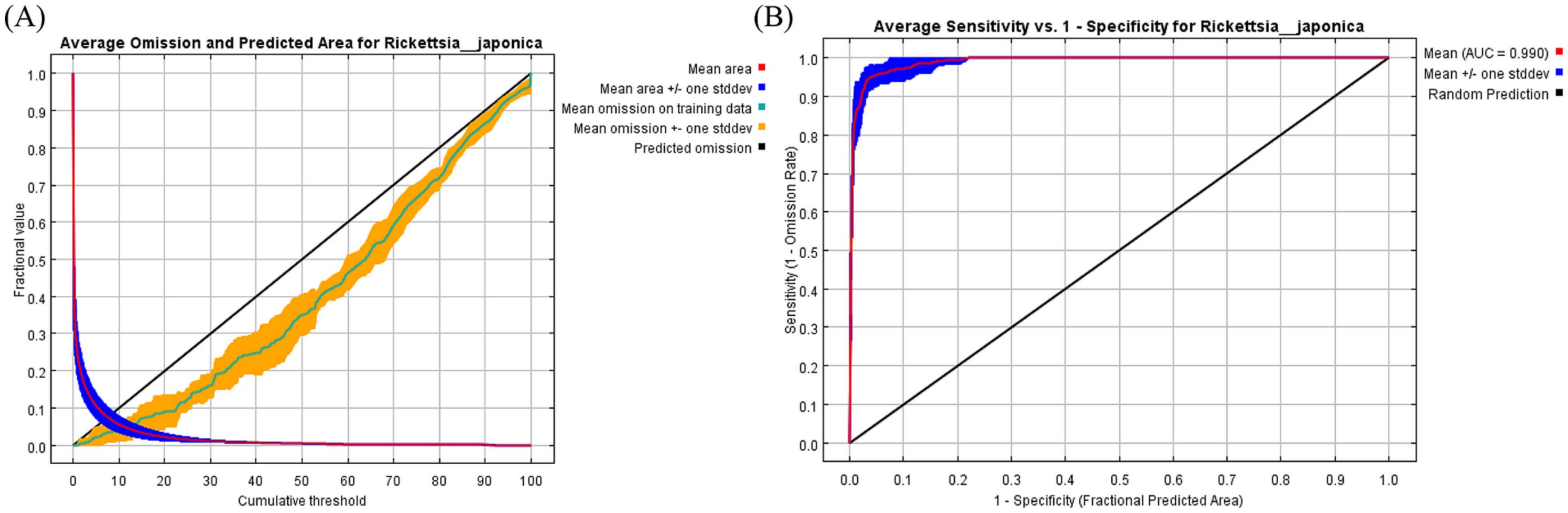
Validation charts of model performance. **(A)** Training omission rate graph, **(B)** receiver operating characteristic curve.

**Figure 2:**
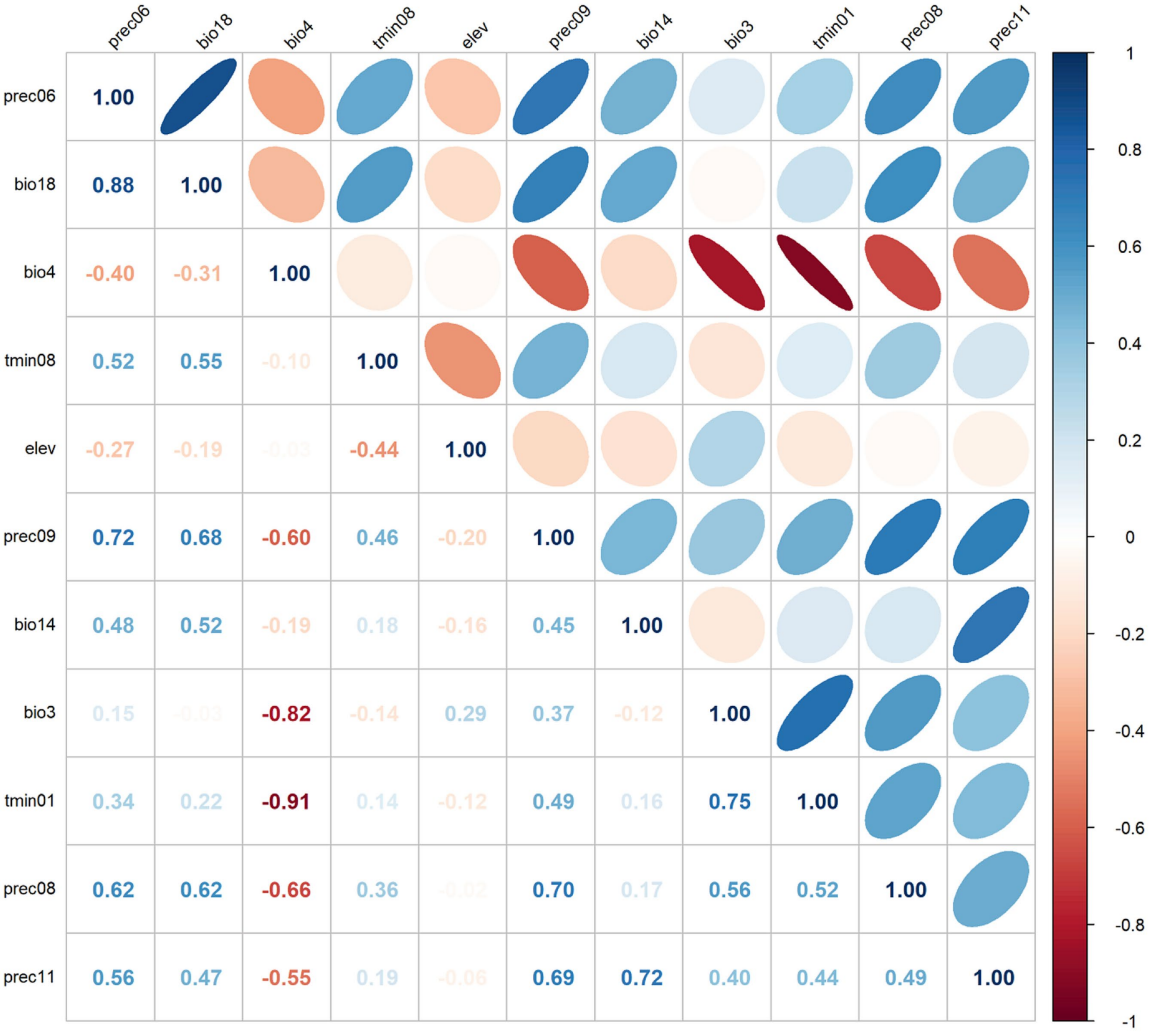
Heat map of Pearson’s correlation coefficient.

In this study, three common metrics, AUC, TSS, and Kappa, were selected as the basis for the assessment of model accuracy. We obtained an AUC value of 0.990, a TSS value of 0.857, and a Kappa value of 0.763 for the model of *R. japonica*. The mean omission of test data of *R. japonica* was in general agreement with the predicted omission (Figure 1A), indicating that the models were well constructed. The AUC value (Figure 1B) was close to 1; The TSS value was >0.85, and the Kappa value was >0.70. According to the model accuracy evaluation reference standards (Table 2), the results of the three evaluation indicators suggest good to excellent levels. These results indicate that the obtained Maxent model predictions were highly accurate. Maxent model revealed that the most influential climatic variables affecting the suitability of *R. japonica*, were average precipitation in June (prec6), precipitation of warmest quarter (bio18),temperature seasonality (bio4), minimum temperature in August (tmin8), elevation (ele), average precipitation in September (prec9), precipitation of driest month (bio14), isothermality (bio3), minimum temperature in January (tmin1), average precipitation in August (prec8), average precipitation in November (prec11). Pearson correlation test was further conducted on the selected climatic variables (Figure 2). Variables exhibiting high correlation but low contribution were subsequently removed, resulting in the retention of eight climatic variables in the final analysis (Table 1).

**Figure 3:**
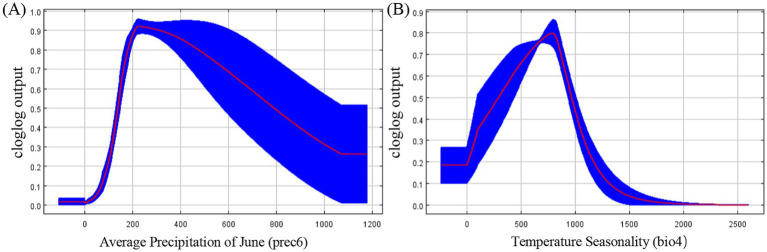
Response curves for dominant climatic factors **(A)** Response curve for average precipitation of June; **(B)** Response curve for temperature seasonality.

Given the substantial contribution rates of prec6 and bio4, we conducted a univariate analysis to elucidate their individual influences. As depicted in Figure 3A, the probability of *R. japonica* presence exhibited an initial increase followed by a subsequent decrease with the rise in Average Precipitation in June. Notably, within the 160–600 mm interval of Average Precipitation in June, the probability exceeded 0.7, indicating highly suitable range. The probability reached its peak value (approximately 0.9) when the Average Precipitation in June was around 200 mm. Furthermore, the Temperature Seasonality variation coefficient within highly suitable habitats ranged from 600 to 900, with a peak around 800, as illustrated in Figure 3B.

**Figure 4:**
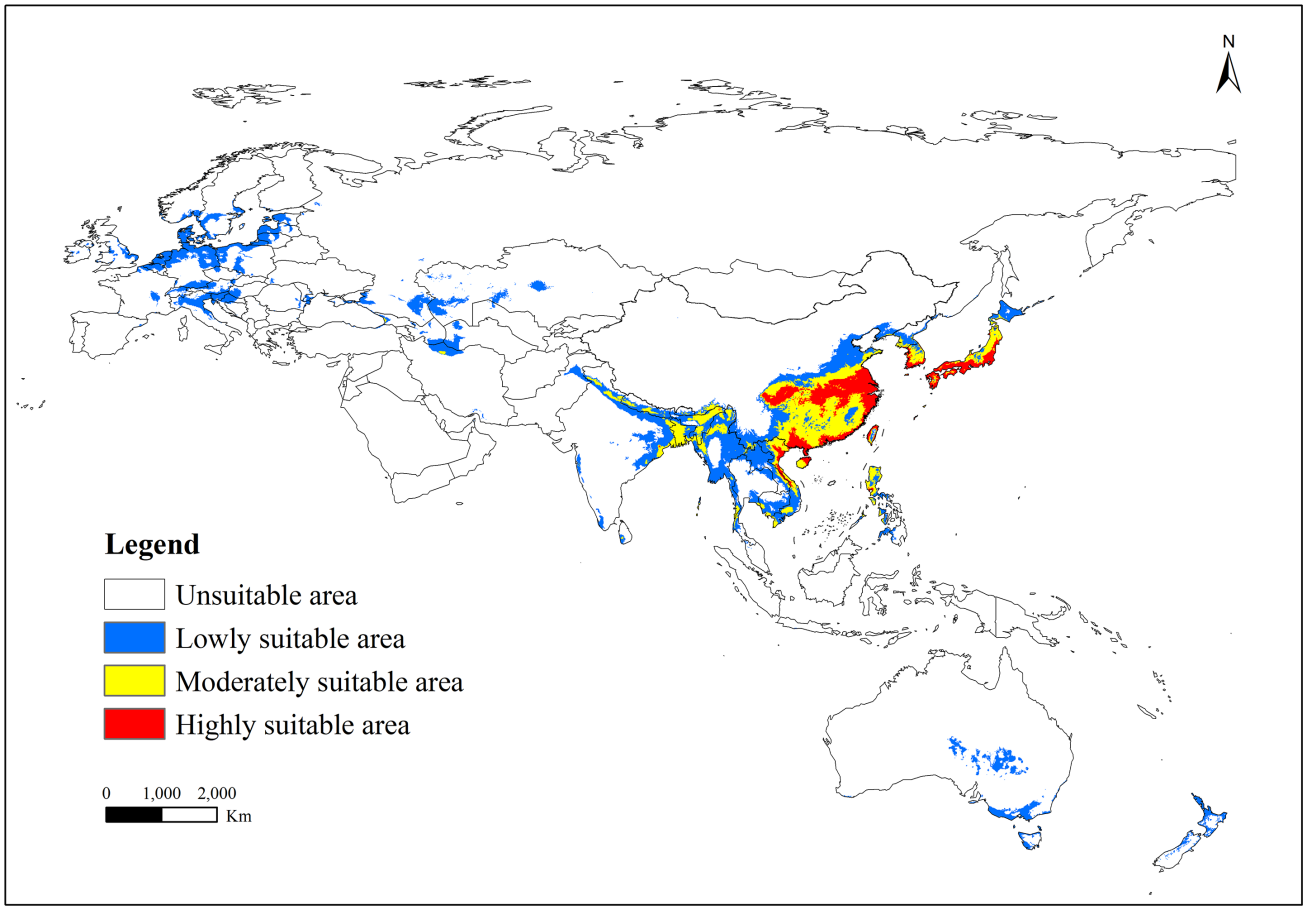
Suitable areas of *R. japonica* under near-current climate conditions.

The distribution of *R. japonica* occurrence points was predominantly in East Asia, Southeast Asia, and several European countries (Figure 4). The predicted potentially suitable area for *R. japonica* under historical climate conditions spanned 17.67 million square kilometers. Within these suitable regions, the proportions of highly suitable, moderately suitable, and less suitable areas were 7.05, 14.64, and 78.31%, respectively. Highly and moderately suitable areas were primarily concentrated in Eastern Asia (including the southeast of China, Korea, and Japan), and Southern Asia (encompassing the east of India, Thailand, Cambodia, Myanmar, Laos, Bangladesh, Vietnam, and the Philippines). Marginally suitable areas were observed not only in the vicinity of moderately and highly suitable regions but also in Europe (including Norway, United Kingdom, Netherlands, Belgium, France, Germany, Switzerland, Austria, Latvia, Estonia, Lithuania, Sweden, Poland, Czech Republic, Italy, Slovakia, Hungary, Croatia, Bosnia and Herzegovina, Ukraine, Romania, Russia, Kazakhstan), in the middle and south of Australia and New Zealand.

**Figure 5:**
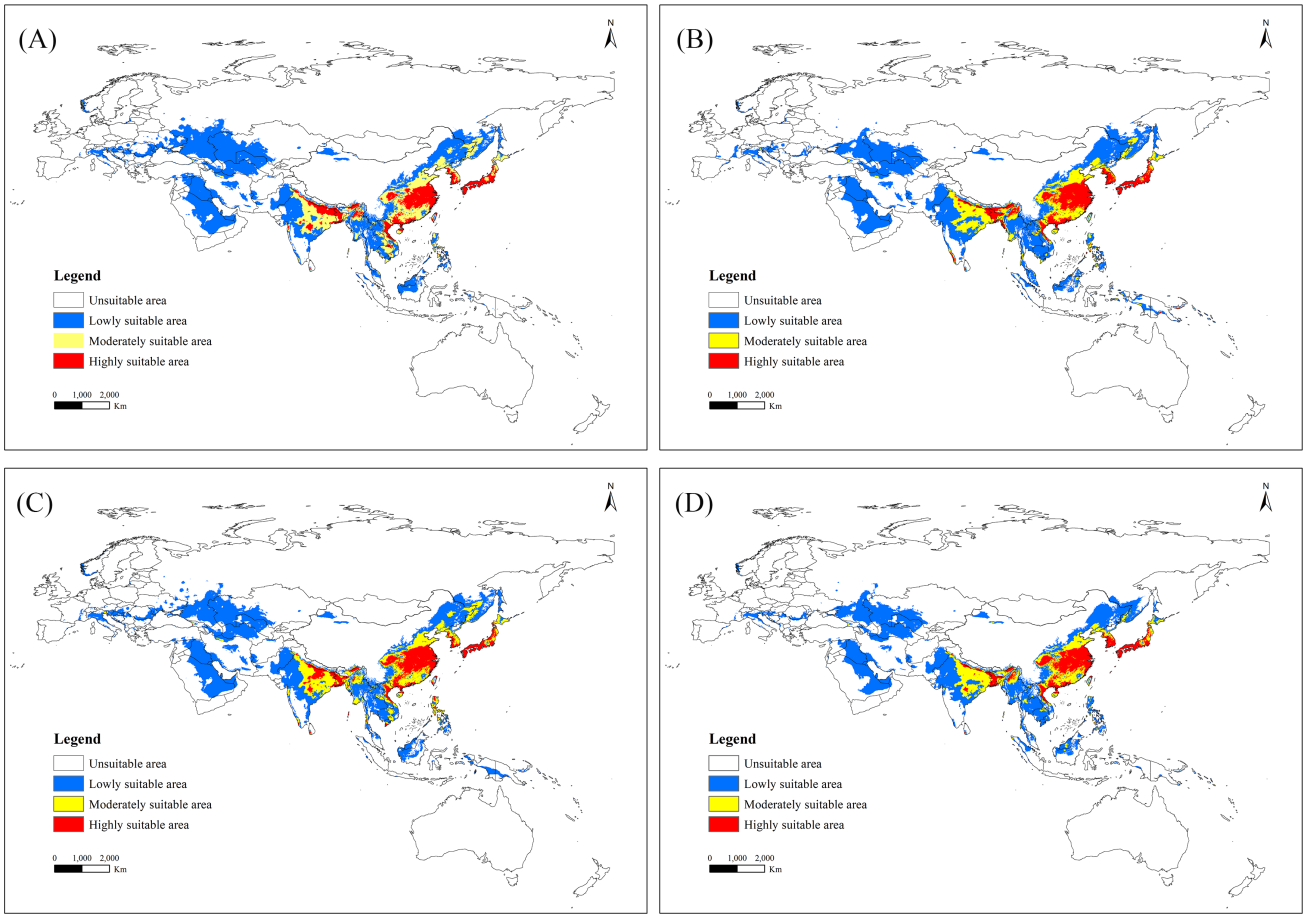
Suitable areas for *R. japonica* in Asia and adjacent regions under future climate scenarios. **(A)** SSP585: 2021–2040; **(B)** SSP370: 2041–2060; **(C)** SSP126: 2061–2080; **(D)** SSP126: 2081–2100.

Among 16 future climate scenarios, the potential range of middle to high suitable areas for *R. japonica* expanded, encompassing regions such as China, Bhutan, Bangladesh, India, Vietnam, Laos, Burma, Cambodia, Thailand and other countries in Southeast Asia (Figure 5). Under the SSP5-8.5 scenario model, the projected maximum extent of the suitable area for Asia and its adjacent regions is anticipated to reach 1525.51 × 10^4^ km^2^, with an area change rate of 118.65% from 2021 to 2040. The maximum area of high suitability for *R. japonica* across different time periods is observed in the SSP3-7.0 scenario for the years 2041–2060, where the high suitability area reached its peak at 193.42 × 10^4^ km^2^, compared to the current suitable area of 95.13 × 10^4^ km^2^ (Table 3).

**Table 3:** Habitat distribution of *R. japonica* in Asia and adjacent regions under current and future climate conditions (×10^4^ km).

Period	Climate scenarios	Total area	Low suitability	Medium suitability	High suitability	Area change	Area changes rate (%)
1970–2000	Current	697.71	425.79	176.78	95.13	–	–
2021–2040	ssp1-2.6	1488.13	1021.31	285.99	180.83	790.42	113.29
	ssp2-4.5	1386.85	945.97	259.18	181.70	689.14	98.77
	ssp3-7.0	1367.48	940.54	253.41	173.53	669.77	96.00
	ssp5-8.5	1525.51	1048.56	295.49	181.46	827.80	118.65
2041–2060	ssp1-2.6	1051.85	647.36	261.37	143.11	354.14	50.76
	ssp2-4.5	1110.86	691.15	249.19	170.53	413.15	59.22
	ssp3-7.0	1430.62	979.42	257.78	193.42	732.91	105.05
	ssp5-8.5	1366.18	932.07	256.92	177.19	668.47	95.81
2061–2080	ssp1-2.6	1437.58	977.09	270.68	189.81	739.87	106.04
	ssp2-4.5	1007.57	572.85	268.40	166.31	309.86	44.41
	ssp3-7.0	1315.24	867.56	269.14	178.54	617.53	88.51
	ssp5-8.5	1388.65	924.94	290.19	173.53	690.94	99.03
2081–2,100	ssp1-2.6	1260.80	867.56	225.62	167.61	563.09	80.71
	ssp2-4.5	1207.46	798.81	260.83	147.83	509.75	73.06
	ssp3-7.0	1196.82	735.62	302.26	158.94	499.11	71.54
	ssp5-8.5	848.71	488.64	229.85	130.22	151.00	21.64

**Figure 6:**
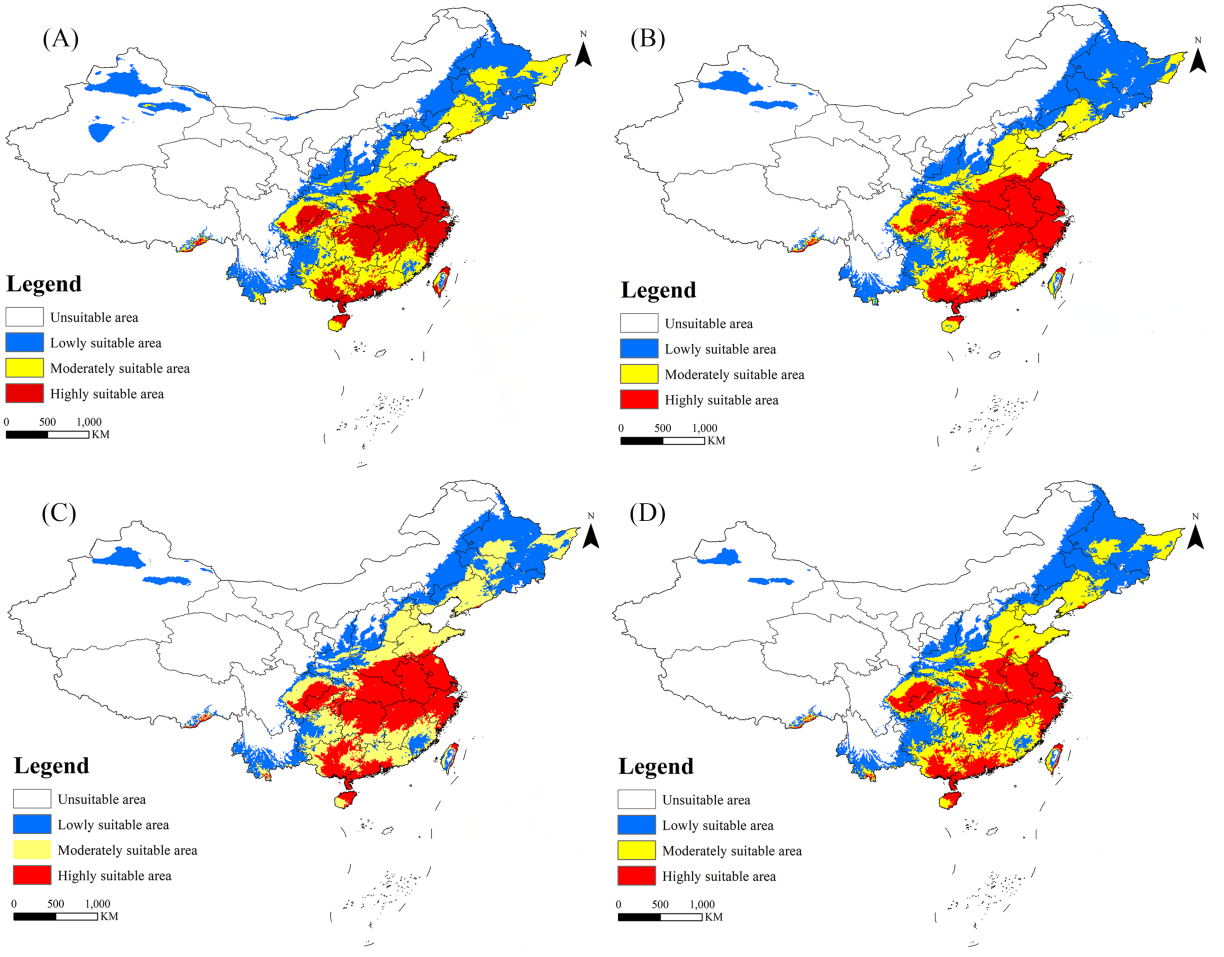
Suitable areas for *R. japonica* in China under future climate scenarios. **(A)** SSP585: 2021–2040; **(B)** SSP370: 2041–2060; **(C)** SSP585: 2061–2080; **(D)** SSP370: 2081–2100.

In the historical climate scenario, China’s suitable areas are primarily concentrated in the southeast coastal regions, as well as the cities along the Qinling Mountains and the Huai River. Moderately and less suitable areas are predominantly found in Liaoning, Hebei, Tianjin, Beijing, Shandong, Shaanxi, and Yunnan. Highly suitable areas are distributed across Henan, Jiangsu, Sichuan, Chongqing, Hubei, Anhui, Hunan, Jiangxi, Zhejiang, Guangdong, Guangxi, Fujian, Hainan, and Taiwan (Figure 6). Under the SSP5-8.5 scenario models, the suitable area of *R. japonica* in China during 2021–2040 is the largest, which is 3.95 × 10^6^ km^2^, and the area change rate is 50.42%. The areas potentially suitable for *R. japonica* under each climate scenario demonstrate an overall increasing trend in the future (Table 4), with suitability areas gradually expanding northward.

**Table 4:** Habitat distribution of *R. japonica* in China under current and future climate conditions (×10^4^ km).

Period	Climate scenarios	Total area	Low suitability	Medium suitability	High suitability	Area change	Area change rate (%)
1970–2000	Current	262.51	77.97	109.41	75.14	–	–
2021–2040	ssp1-2.6	359.83	129.35	117.70	112.77	97.32	37.07
	ssp2-4.5	355.94	142.40	112.78	100.76	93.43	35.59
	ssp3-7.0	351.56	148.79	103.78	98.98	89.05	33.92
	ssp5-8.5	394.86	155.27	137.63	101.96	132.35	50.42
2041–2060	ssp1-2.6	366.38	136.02	130.28	100.08	103.87	39.57
	ssp2-4.5	362.54	130.23	121.08	111.23	100.03	38.11
	ssp3-7.0	379.06	148.95	110.64	119.47	116.55	44.40
	ssp5-8.5	351.15	135.77	127.44	87.94	88.64	33.76
2061–2080	ssp1-2.6	358.06	130.40	112.00	115.66	95.55	36.40
	ssp2-4.5	367.45	125.52	133.40	108.53	104.94	39.98
	ssp3-7.0	370.98	137.76	128.46	104.76	108.47	41.32
	ssp5-8.5	373.14	131.37	130.42	111.35	110.63	42.14
2081–2,100	ssp1-2.6	346.85	137.29	98.51	111.05	84.34	32.13
	ssp2-4.5	352.05	142.68	114.86	94.51	89.54	34.11
	ssp3-7.0	375.37	141.24	131.02	103.11	112.86	42.99
	ssp5-8.5	358.13	157.22	119.84	81.07	95.62	36.43

## 4 Discussion

We employed the Maxent model with optimized parameters to forecast the future suitable areas of *R. japonica* Asian and adjacent regions under four SSPs scenarios, serving as an early warning. Our findings indicate that mean precipitation in June, temperature seasonality, minimum temperature in August, and elevation are the primary factors influencing the potential geographical distribution of *R. japonica*, with mean precipitation in June exhibiting the greatest contribution rates and permutation importance. The projected suitable areas of *R. japonica* in different periods under each climate scenario, both Asian and within China, surpass those under the current climate scenario. In Asian and adjacent regions, Japan emerges as the most favorable region for *R. japonica*. In Southeast Asia, suitable zones transition from moderate-low to moderate-high suitability. Moreover, potential suitable zones in Europe show signs of expansion. Under future climate scenarios, the highly suitable areas for *R. japonica* in central and southern China are projected to increase, with the suitable areas gradually expanding northward.

Currently, there were few literatures exploring the relationship between climate and JSF. The majority of research were concentrated on clinical symptoms of cases and serological testing (9, 40–43). According to the latest monitoring data from Japan, the incidence rate of JSF had been consistently rising until 2016 (44). It was observed that JSF had become more prevalent among the older adult population by 2020. More importantly, the incidence of JSF is increasing in areas not previously considered high-incidence areas (45). We suspect that this phenomenon is related to changes in temperature and increased precipitation. It was predicted that Tick-Borne Diseases (TBD) would become prevalent in new regions due to global warming, prompting ticks that prefer warmer environments to become more active (17, 46). As observed in mainland China, climate factors such as temperature, sunshine duration, precipitation, and seasonal indicators are correlated with the incidence rate of TBD (19). Our research has also found the average precipitation in June, temperature seasonality, and the minimum temperature in August contributed strongly to the distribution of *R. japonica*. In regions with high precipitation and relatively high temperatures, the suitable habitat area for *R. japonica* is larger. These findings were consistent with a study conducted in Japan, which revealed that the incidence rate of JSF is related to year-round seasonal temperature fluctuations (47). The possible reason for this is the continuous increase in the population size of the vector ticks, allowing them to attack hosts even during winter. The prevalence of *R. japonica* is mainly from April to December, and the vector ticks activity shift with the change of climate, which can attack the hosts at the end of calendar winter and in early spring (48).

In southern China, the distribution areas of *R. japonica* share the same Köppen climate classification, known as Cfa, characterized by a temperate climate with hot summers and an absence of dry seasons. This climate type entails a minimum monthly mean temperature ranging from 0 to 18°C and a maximum monthly mean temperature of 22°C or higher throughout the year, coupled with high humidity (31). Over the past 40 years, the Cfa climate classification has notably expanded in China, mirroring the findings of our study, where highly suitable areas for *R. japonica* have demonstrated a tendency to expand in southern China. Moreover, within suitable areas of China, *R. japonica* has expanded northward. This expansion can primarily be attributed to the projected warmer and wetter climate in the North, which enhances the climate suitability for tick survival in many regions (49, 50).

The expansion of the suitable area implies an increasing public health risk for the population residing within these regions. We unexpectedly found that the incidence of JSF is increasing in eastern Japan (Fukushima, Ibaraki, Tochigi, Gunma, and Shizuoka Prefectures), where the disease has previously been regarded as non-prevalent regions (51, 52). Meanwhile, reports of JSF cases in China have also gradually increased over the past 5 years (53–56). Therefore, it is imperative to enhance health education regarding tick prevention and control measures among the population residing in the potentially suitable areas, disseminating knowledge about the course and etiology of JSF among healthcare professionals as well as in society (57). Simultaneously, promoting and guiding individuals to take protective measures to prevent tick contact and bites. This includes using repellents, wearing untreated or permethrin-treated protective clothing, and conducting tick checks after entering indoor spaces, followed by showering to assist in detecting ticks on the skin (58). In addition, regular monitoring of tick density and activity, assessing the potential health risks posed by ticks to humans and animals, effective prevention through environmental modification, biological control, and physical barriers, developing an early warning system for emerging tick-borne diseases, and conducting targeted vaccination campaigns in high-incidence areas (59).

Our research presents distinct advantages. Firstly, there is currently no existing study on the risk prediction of *R. japonica* and JSF. This study introduces a systematic approach for Asian and adjacent regions predicting the distribution of *R. japonica*. The Maxent model, based on the principle of maximum entropy, offers several advantages: it exhibits higher accuracy in prediction outcomes and is capable of achieving superior modeling results with smaller sample sizes, which is importantly notable (60, 61). Additionally, Maxent facilitates the identification and interpretation of non-linear responses. Contributions and Jackknife analyses promptly highlight crucial variables, while response curves illustrate the variation in probabilities with dependent variables, providing comprehensive information (62). Maxent further excels over other models such as GLMs, GAMs, BIOCLIM, and GARP in predictive accuracy due to its ability to model complex environmental variables (63). Furthermore, predictions were enhanced by overlaying coordinates of *H. longicornis* and *R. japonica*, significantly improving the accuracy of risk distribution forecasts. This approach identifies numerous prospective regions susceptible to *R. japonica* invasion in China. While not yet intercepted or detected in China, these regions could potentially be colonized under specific conditions. Lastly, the expansion of suitable areas for *R. japonica* towards northern China is attributed to global warming and the significant increase in the Cfa climate, highlighting a notable trend in our findings.

Several limitations were identified in our study that highlight areas for improvement in future research. Firstly, our reliance on historical climate data spanning from 1970 to 2000 restricted access to current climate information, potentially impacting the accuracy of current suitability estimates. Moreover, the exclusive use of the Maxent model focused solely on species’ climatic adaptability and specific climatic variables, neglecting the inclusion of other critical factors. To enhance precision in estimating distribution within suitable habitats, future studies should incorporate a broader range of variables including soil moisture levels, vegetation cover types, population densities, tick vector densities, and land use patterns. Finally, and of critical importance, due to the limitations of current data, the geographical scope for future predictions is limited to analysis of Asia and adjacent regions. Although this study has enhanced model performance by refining feature selection and adjusting regularization parameters to optimize model fitting, it is imperative to exercise caution when extrapolating the model. Future research should focus on collecting more data from various regions to further validate and refine the model.

## 5 Conclusion

Detecting potential regions suitable for species invasion holds significant importance in preventing alien incursions. Utilizing the Maxent model, we evaluated the potential invasion ranges of *R. japonica* under both current and future climate conditions. Our findings reveal that the potential invasion range of *R. japonica* predominantly spans subtropical and warmer temperate regions, encompassing areas such as Japan, South Korea, southeast China, various southeast Asian countries, and European nations. Temperature seasonality and precipitation emerged as pivotal climatic factors influencing the potential distribution of *R. japonica*. Looking ahead, the invasion range of *R. japonica* is poised to expand globally. Within China, the southeastern region witnesses an increase in high-suitability areas, with a discernible trend of expansion towards the north. These insights furnish valuable reference points for devising appropriate management strategies aimed at thwarting the establishment and further dissemination of *R. japonica* throughout the world.

## Data Availability

The original contributions presented in the study are included in the article/Supplementary material, further inquiries can be directed to the corresponding author.

## Supplementary material

SUPPLEMENTARY FIGURE 1 Distribution of data points around the world.
