# Supplementary material for: Projecting the potential distribution of Rickettsia japonica in China and Asian adjacent regions under climate change using the Maxent model
Source: Front Public Health. 2025 Mar 6;13:1478736. doi: 10.3389/fpubh.2025.1478736 (PMC11922925; doi:10.3389/fpubh.2025.1478736)
Supplement: SUPPLEMENTARY FIGURE 1 — Distribution of data points around the world. [file Data_Sheet_1.zip › Supplementary material/Supplementary Table 1.DOCX]

| Supplementary Table 1. Definitions of the Nineteen Bioclimatic Variables |
| --- |
| BIO1 = Annual Mean Temperature |
| BIO2 = Mean Diurnal Range [Mean of monthly (max temp− min temp)] |
| BIO3 = Isothermality [(BIO2/BIO7) × 100] |
| BIO4 = Temperature Seasonality (standard deviation ×100) |
| BIO5 = Max Temperature of Warmest Month |
| BIO6 = Min Temperature of Coldest Month |
| BIO7 = Temperature Annual Range (BIO5− BIO6) |
| BIO8 = Mean Temperature of Wettest Quarter |
| BIO9 = Mean Temperature of Driest Quarter |
| BIO10 = Mean Temperature of Warmest Quarter |
| BIO11 = Mean Temperature of Coldest Quarter |
| BIO12 = Annual Precipitation |
| BIO13 = Precipitation of Wettest Month |
| BIO14 = Precipitation of Driest Month |
| BIO15 = Precipitation Seasonality (Coefficient of Variation) |
| BIO16 = Precipitation of Wettest Quarter |
| BIO17 = Precipitation of Driest Quarter |
| BIO18 = Precipitation of Warmest Quarter |
| BIO19 = Precipitation of Coldest Quarter |
