# Supplementary figures and images for: Projecting the potential distribution of Rickettsia japonica in China and Asian adjacent regions under climate change using the Maxent model
Source: Front Public Health. 2025 Mar 6;13:1478736. doi: 10.3389/fpubh.2025.1478736 (PMC11922925; doi:10.3389/fpubh.2025.1478736)

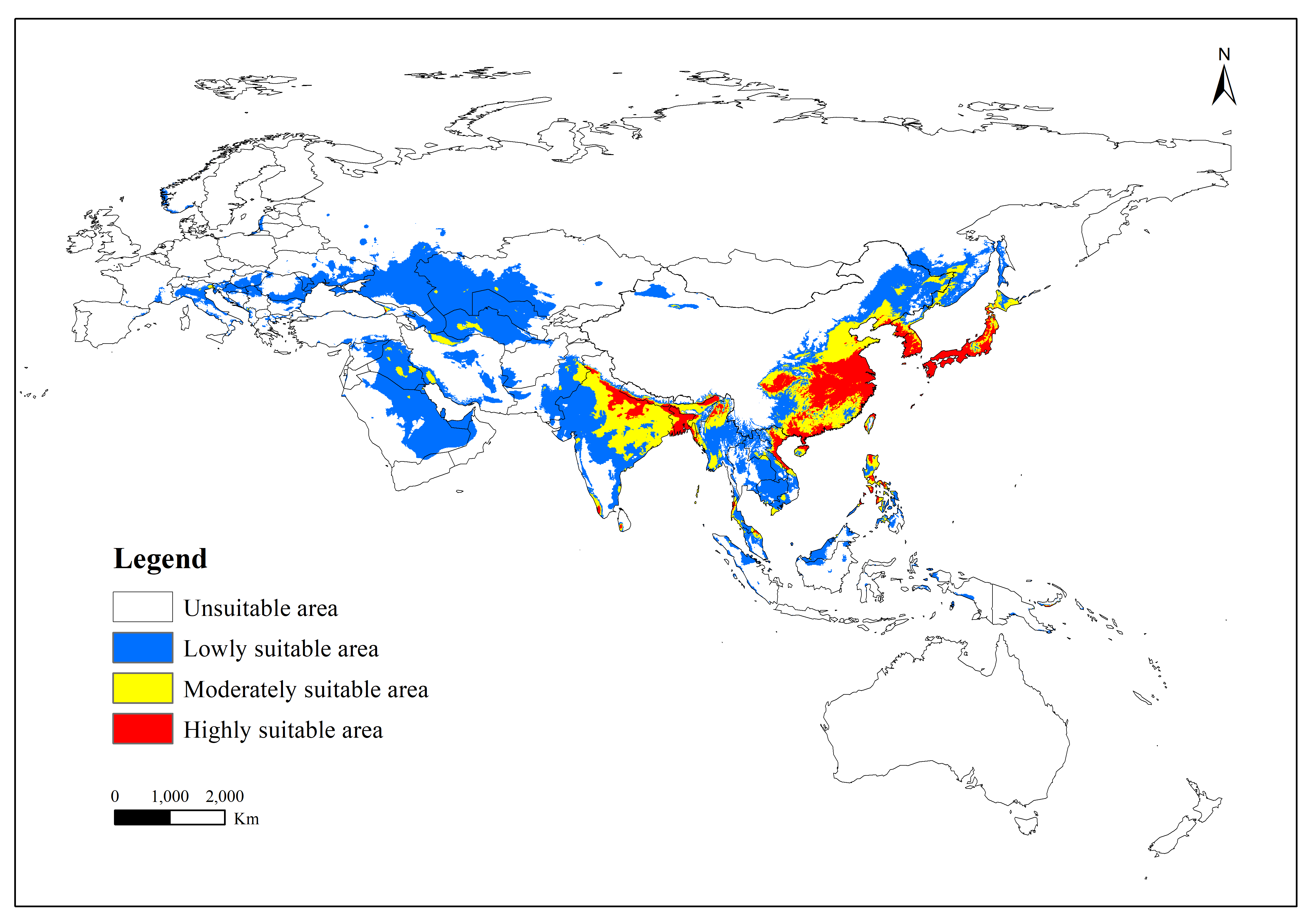

Supplement: SUPPLEMENTARY FIGURE 1 — Distribution of data points around the world. [file Data_Sheet_1.zip › Supplementary material/Supplementary files/Future distribution of Asian and neighboring countries/2021-2040 ssp126.tif]

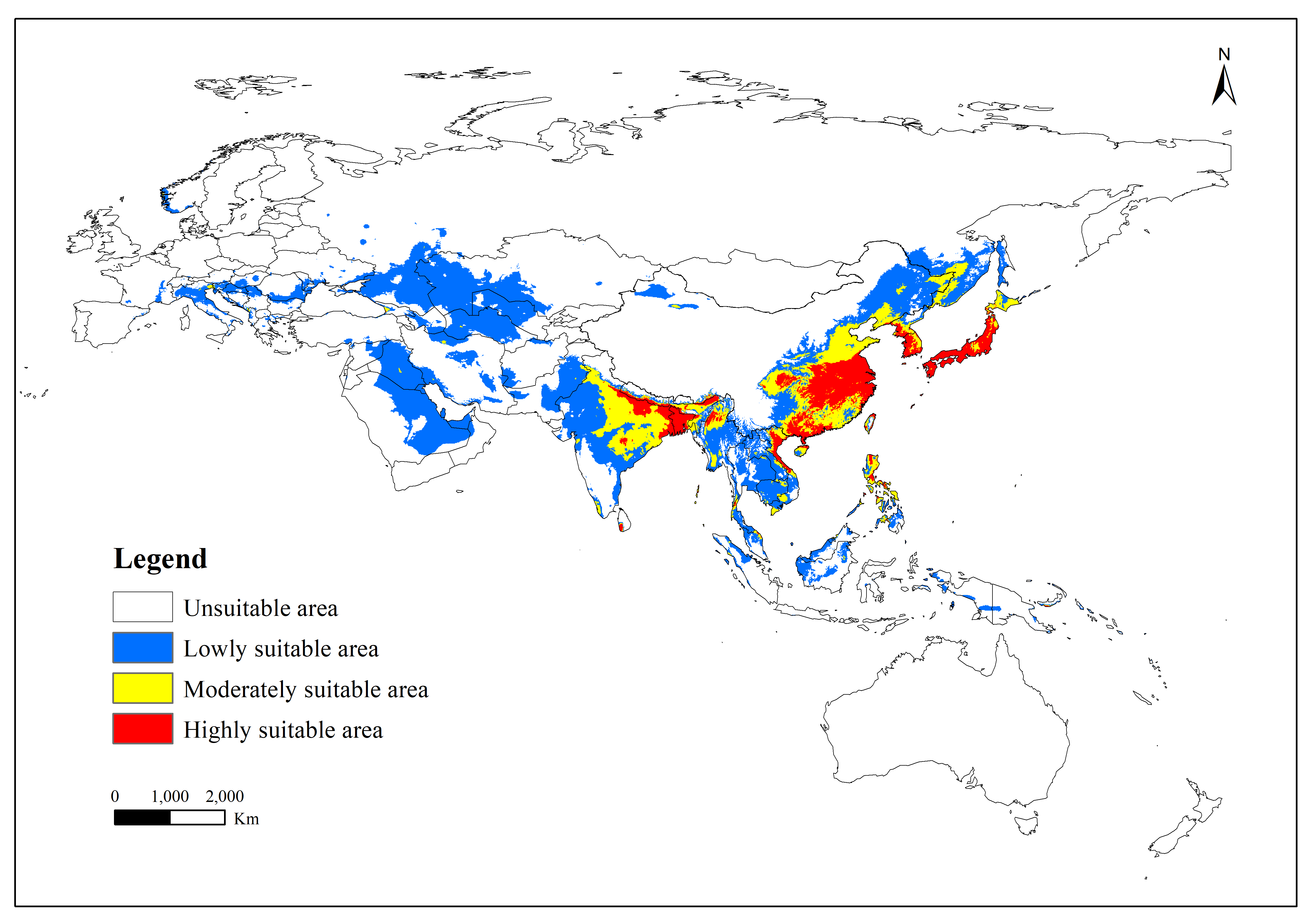

Supplement: SUPPLEMENTARY FIGURE 1 — Distribution of data points around the world. [file Data_Sheet_1.zip › Supplementary material/Supplementary files/Future distribution of Asian and neighboring countries/2021-2040 ssp245.tif]

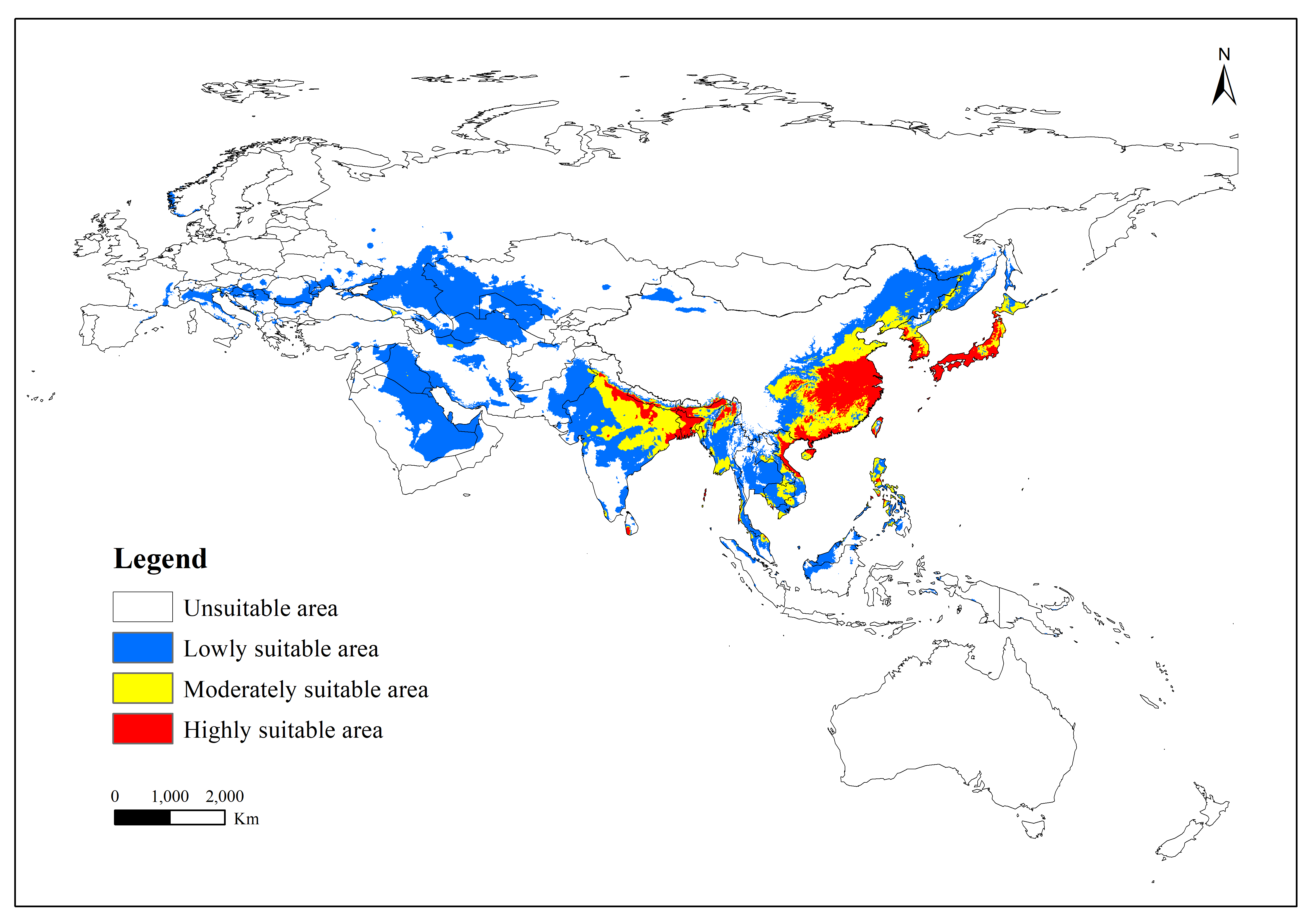

Supplement: SUPPLEMENTARY FIGURE 1 — Distribution of data points around the world. [file Data_Sheet_1.zip › Supplementary material/Supplementary files/Future distribution of Asian and neighboring countries/2021-2040 ssp370.tif]

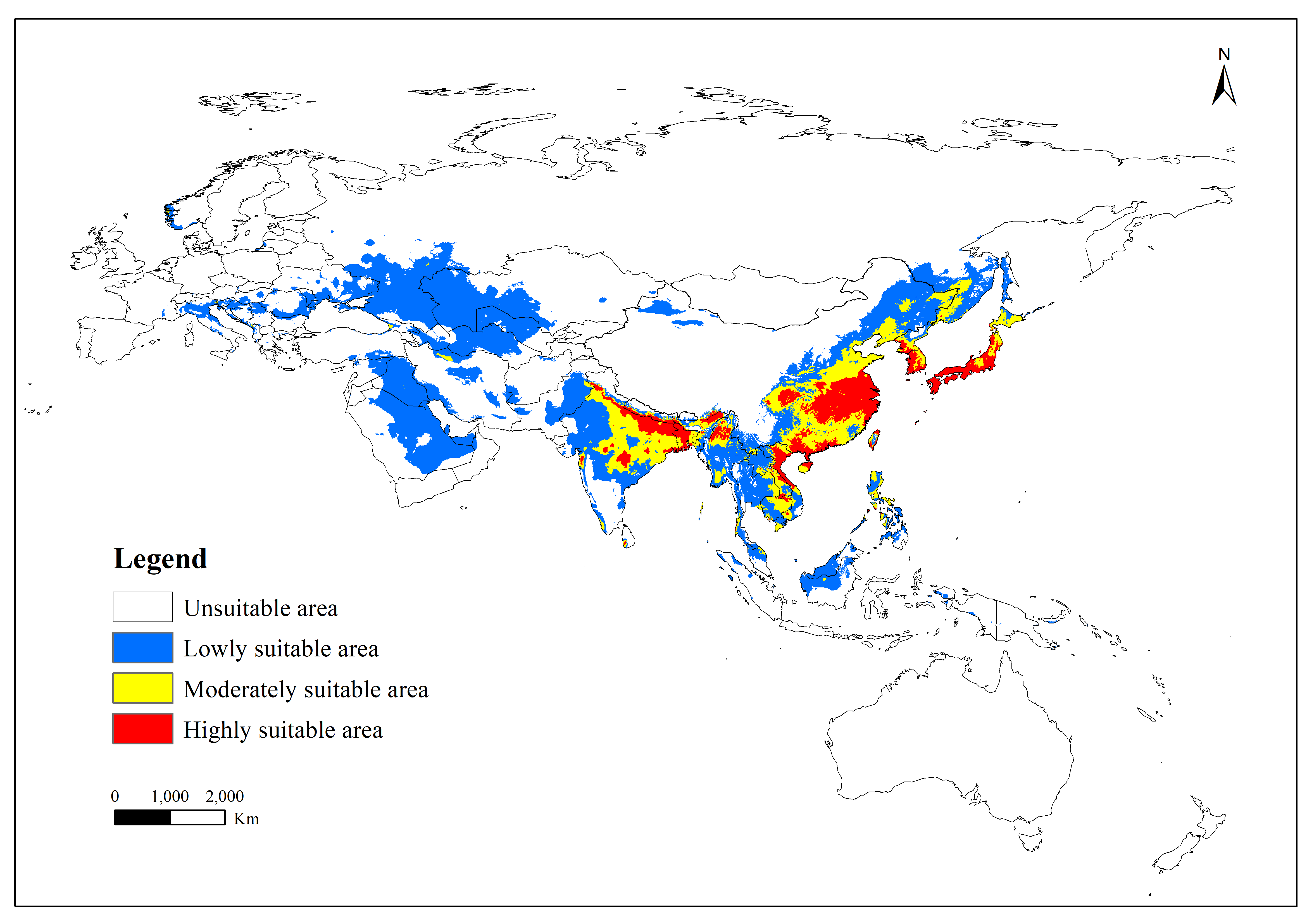

Supplement: SUPPLEMENTARY FIGURE 1 — Distribution of data points around the world. [file Data_Sheet_1.zip › Supplementary material/Supplementary files/Future distribution of Asian and neighboring countries/2021-2040 ssp585.tif]

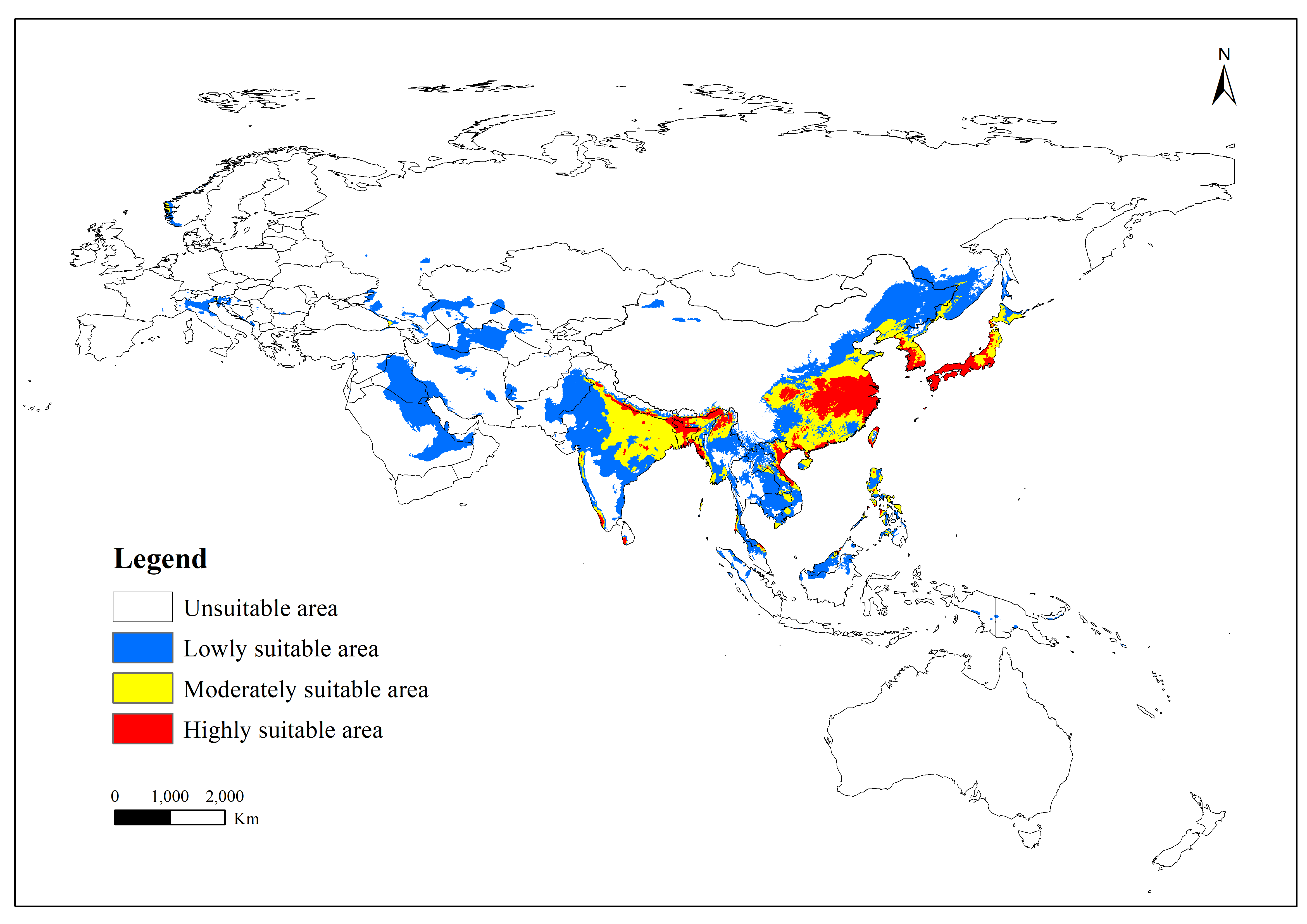

Supplement: SUPPLEMENTARY FIGURE 1 — Distribution of data points around the world. [file Data_Sheet_1.zip › Supplementary material/Supplementary files/Future distribution of Asian and neighboring countries/2041-2060 ssp126.tif]

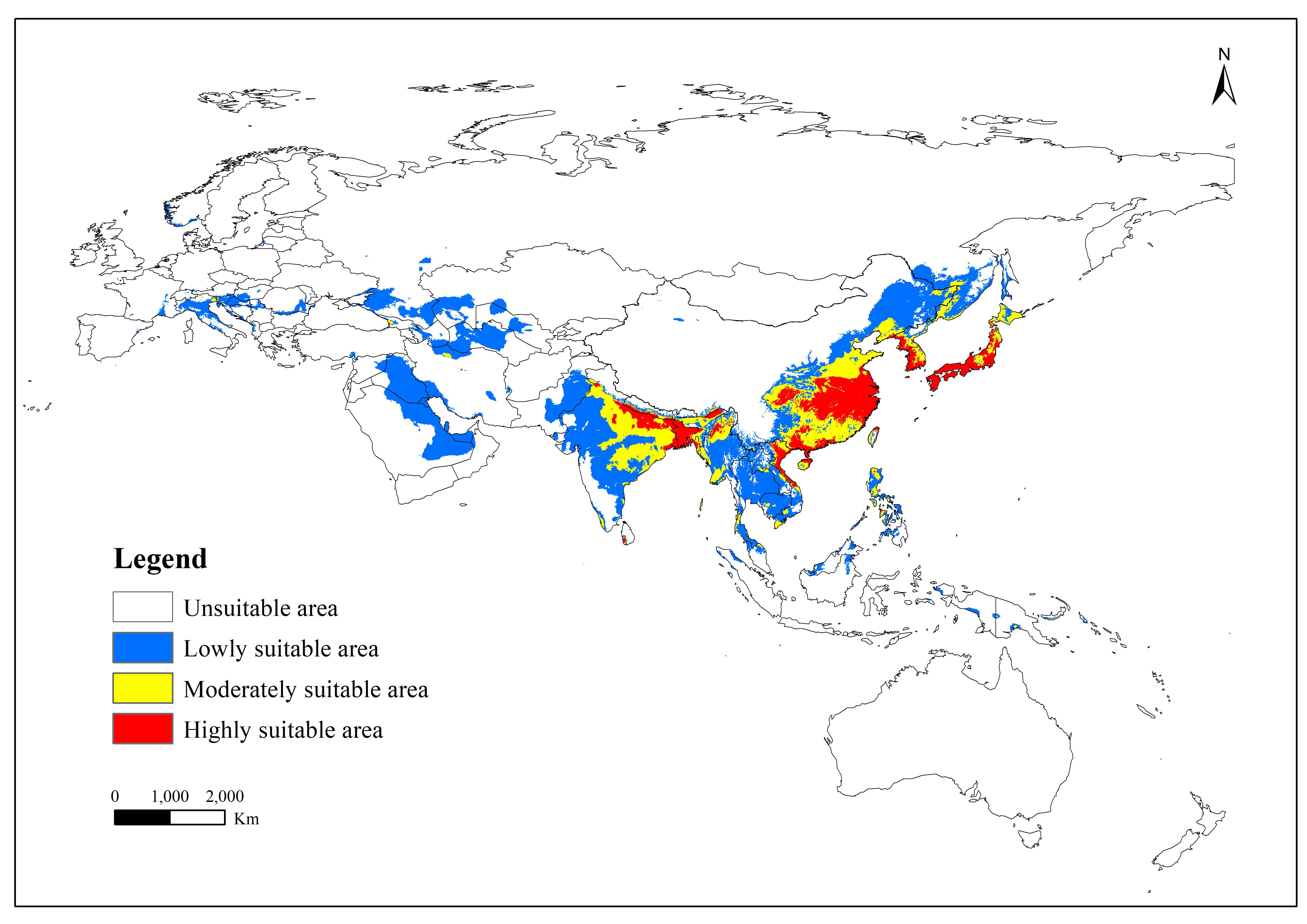

Supplement: SUPPLEMENTARY FIGURE 1 — Distribution of data points around the world. [file Data_Sheet_1.zip › Supplementary material/Supplementary files/Future distribution of Asian and neighboring countries/2041-2060 ssp245.tif]

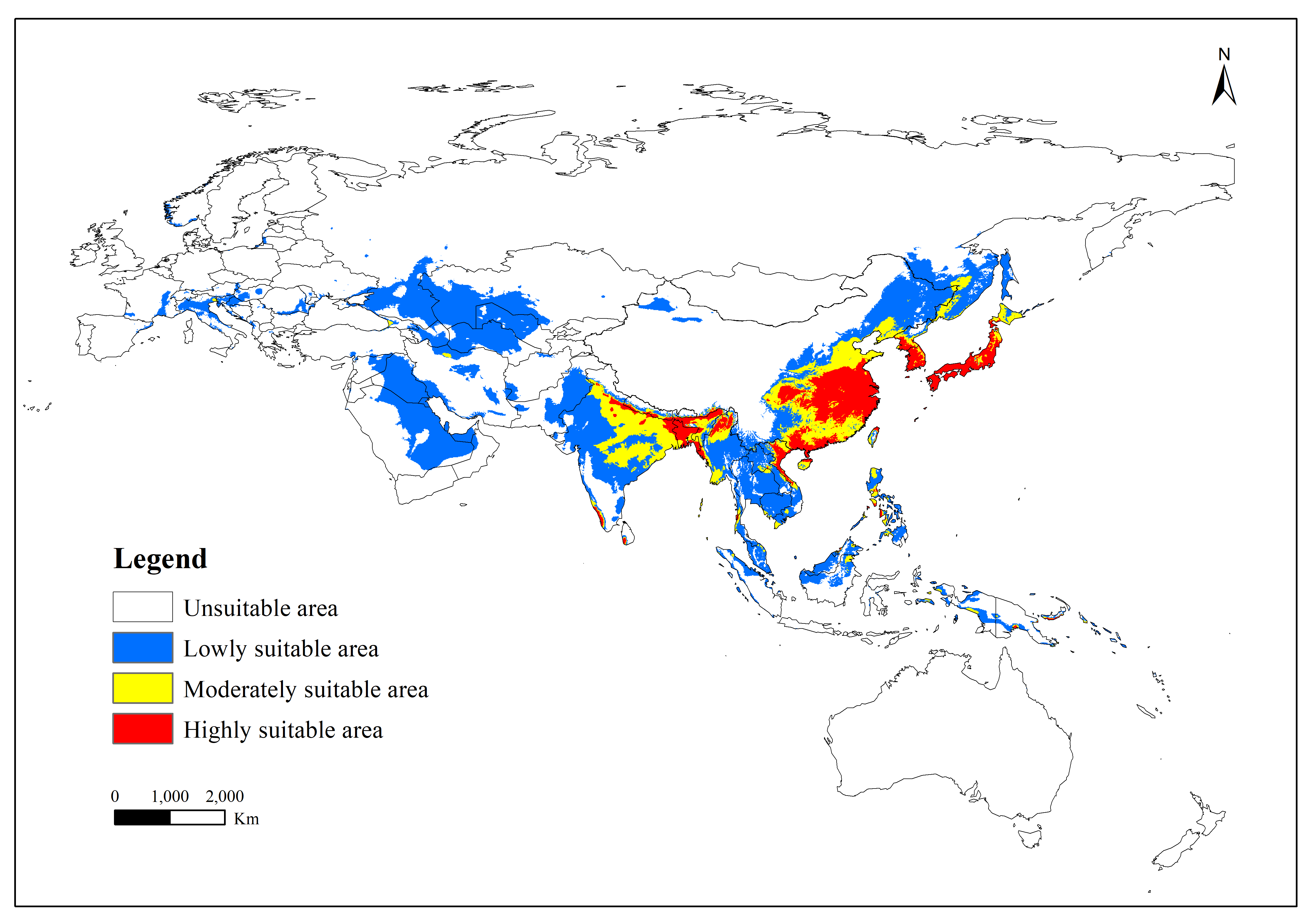

Supplement: SUPPLEMENTARY FIGURE 1 — Distribution of data points around the world. [file Data_Sheet_1.zip › Supplementary material/Supplementary files/Future distribution of Asian and neighboring countries/2041-2060 ssp370.tif]

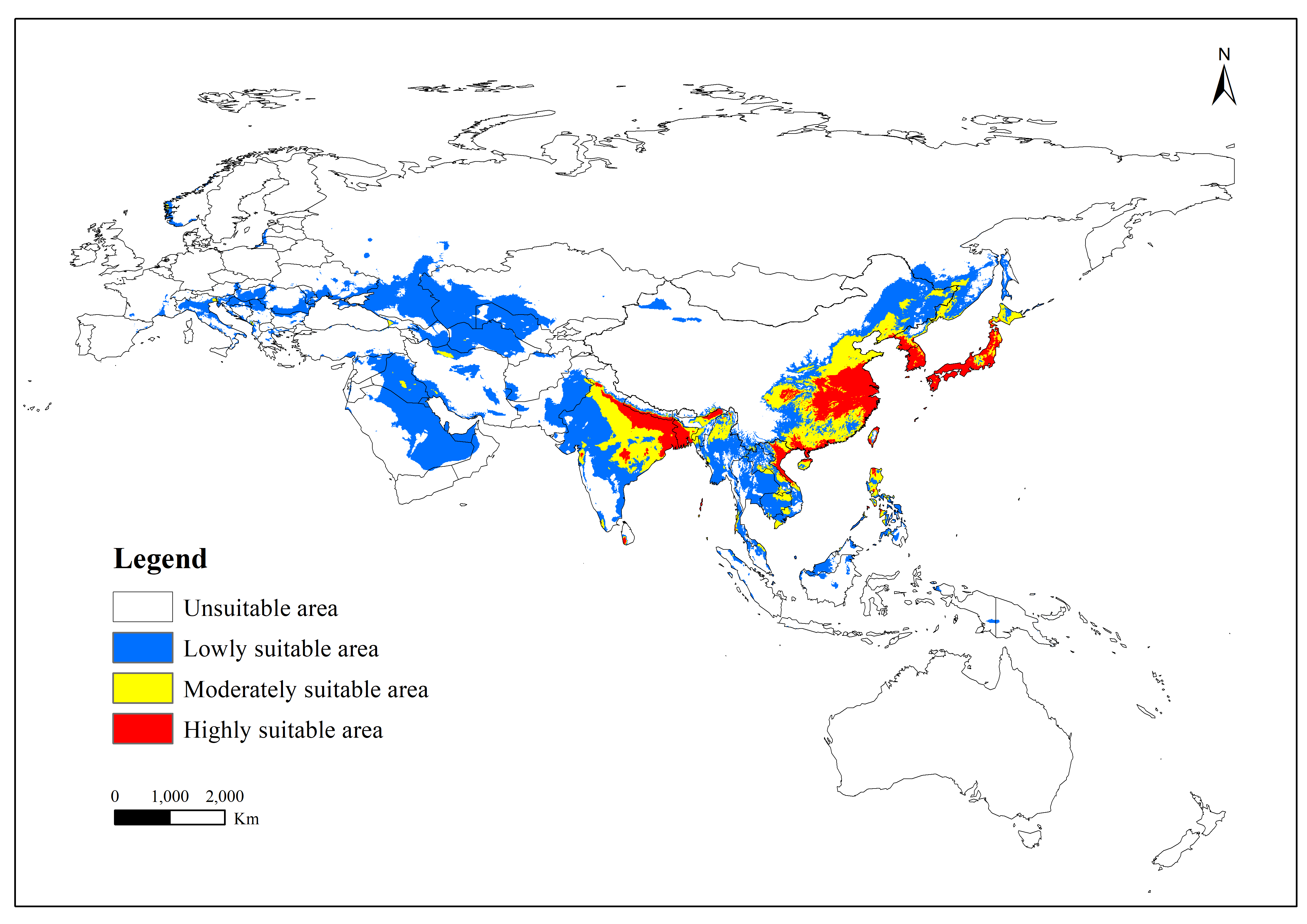

Supplement: SUPPLEMENTARY FIGURE 1 — Distribution of data points around the world. [file Data_Sheet_1.zip › Supplementary material/Supplementary files/Future distribution of Asian and neighboring countries/2041-2060 ssp585.tif]

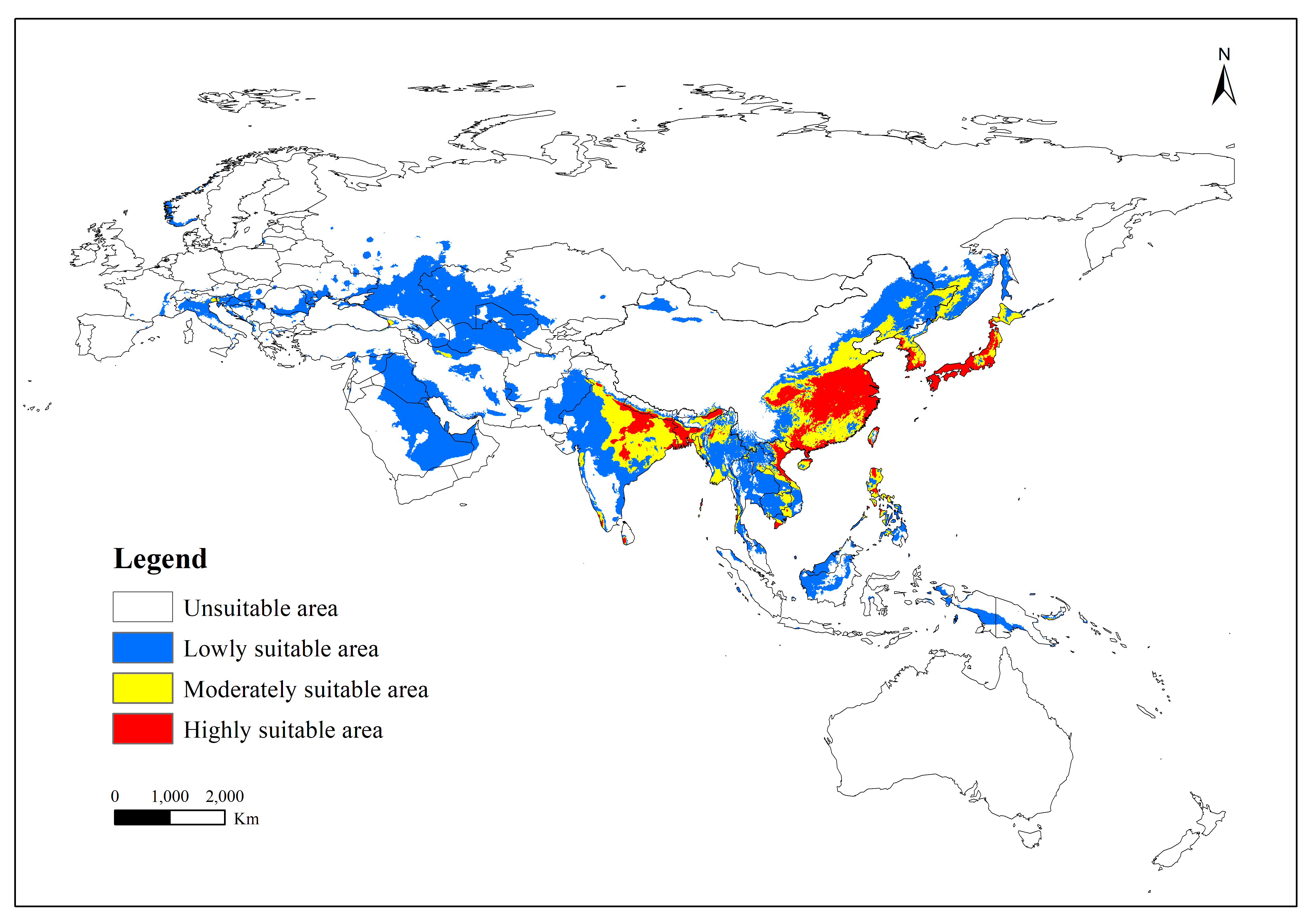

Supplement: SUPPLEMENTARY FIGURE 1 — Distribution of data points around the world. [file Data_Sheet_1.zip › Supplementary material/Supplementary files/Future distribution of Asian and neighboring countries/2061-2080 ssp126.tif]

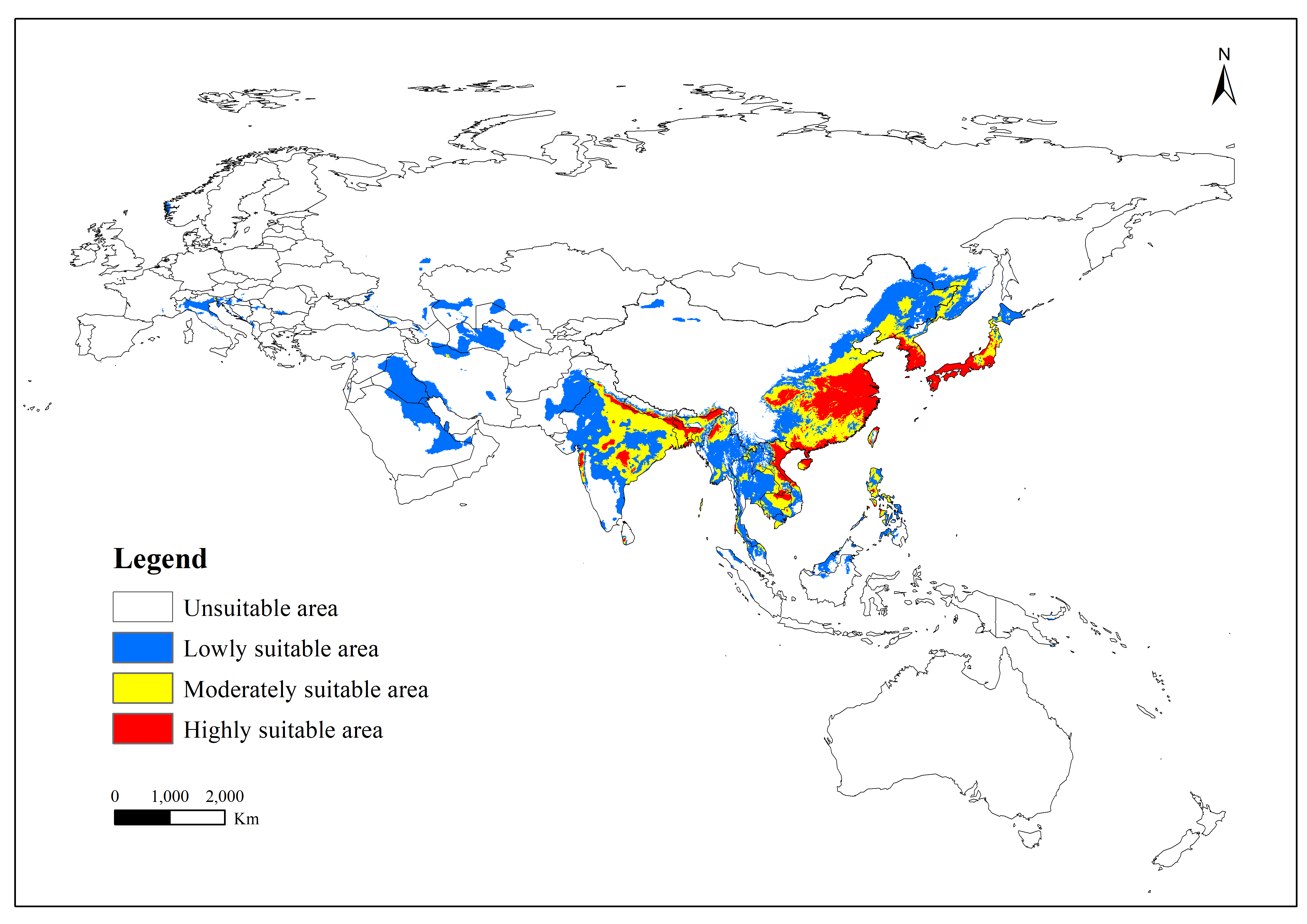

Supplement: SUPPLEMENTARY FIGURE 1 — Distribution of data points around the world. [file Data_Sheet_1.zip › Supplementary material/Supplementary files/Future distribution of Asian and neighboring countries/2061-2080 ssp245.tif]

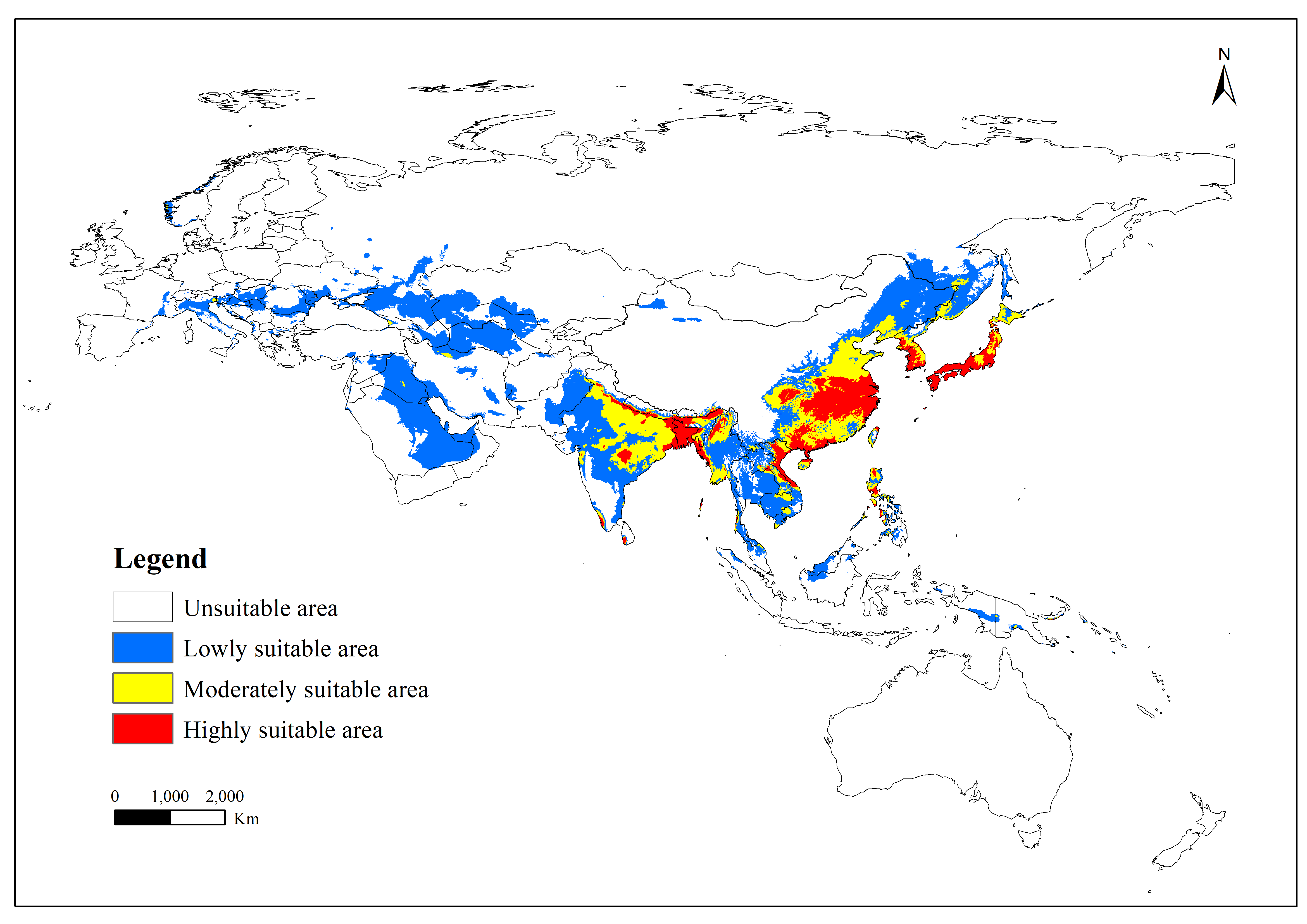

Supplement: SUPPLEMENTARY FIGURE 1 — Distribution of data points around the world. [file Data_Sheet_1.zip › Supplementary material/Supplementary files/Future distribution of Asian and neighboring countries/2061-2080 ssp370.tif]

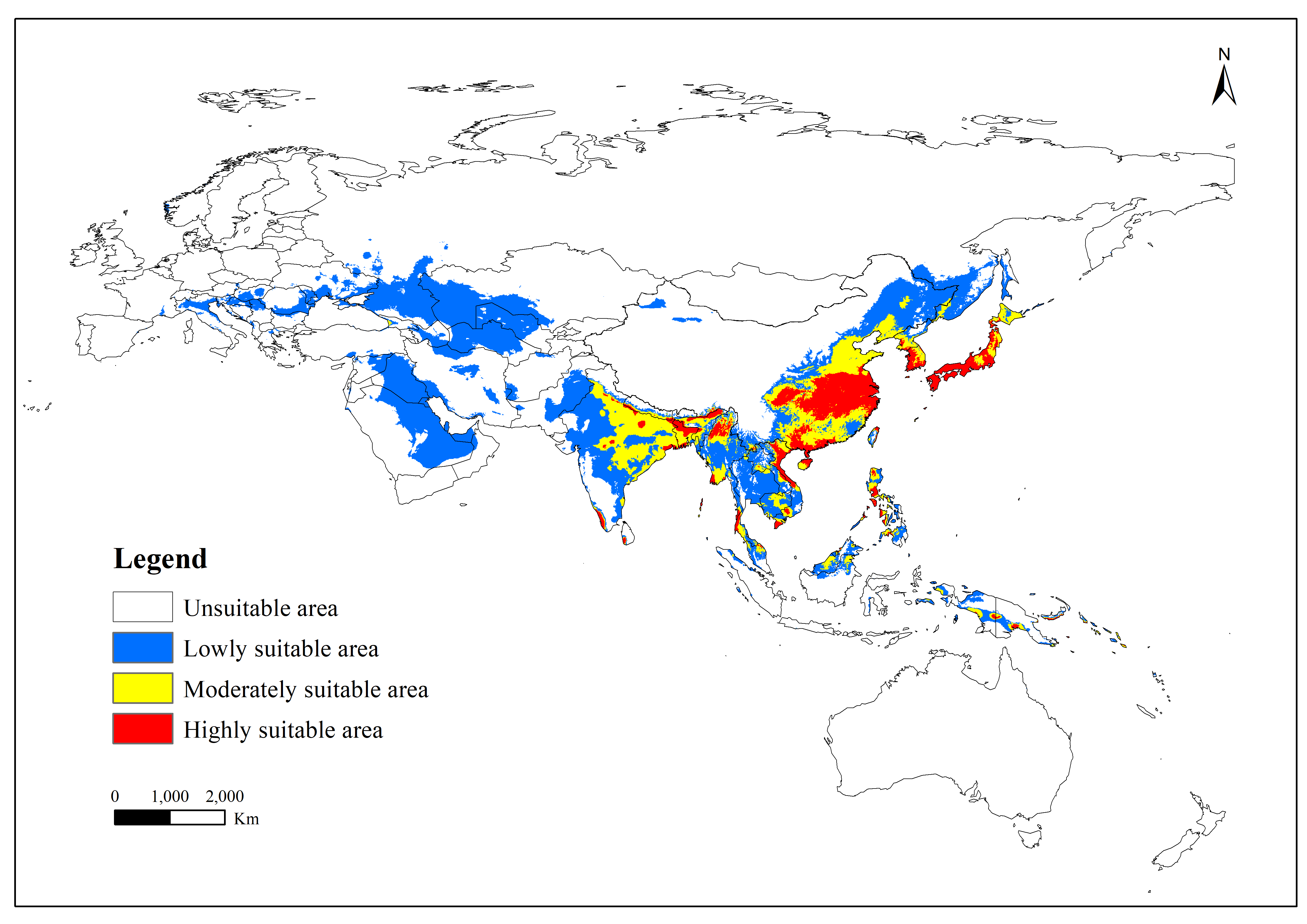

Supplement: SUPPLEMENTARY FIGURE 1 — Distribution of data points around the world. [file Data_Sheet_1.zip › Supplementary material/Supplementary files/Future distribution of Asian and neighboring countries/2061-2080 ssp585.tif]

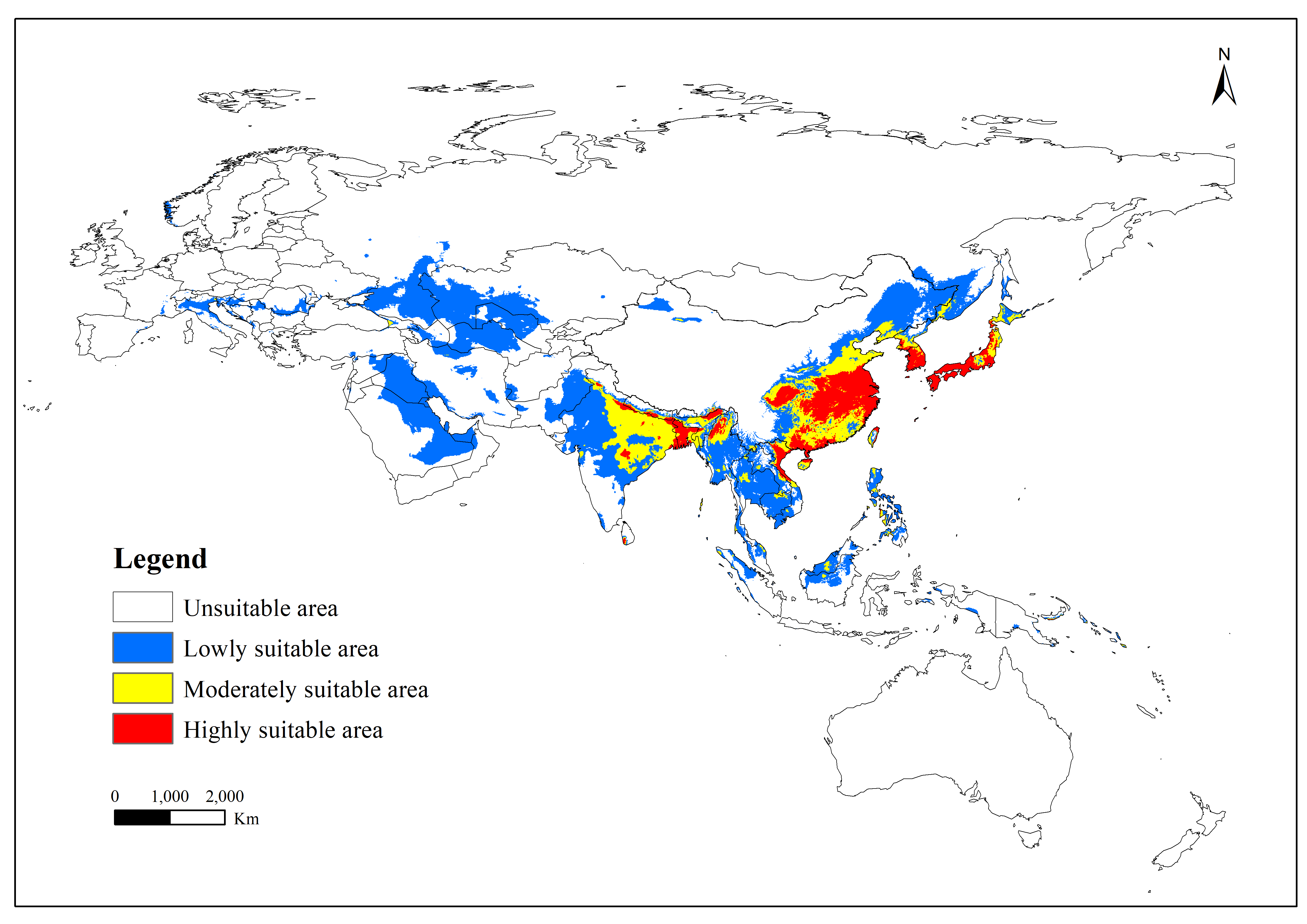

Supplement: SUPPLEMENTARY FIGURE 1 — Distribution of data points around the world. [file Data_Sheet_1.zip › Supplementary material/Supplementary files/Future distribution of Asian and neighboring countries/2081-2100 ssp126.tif]

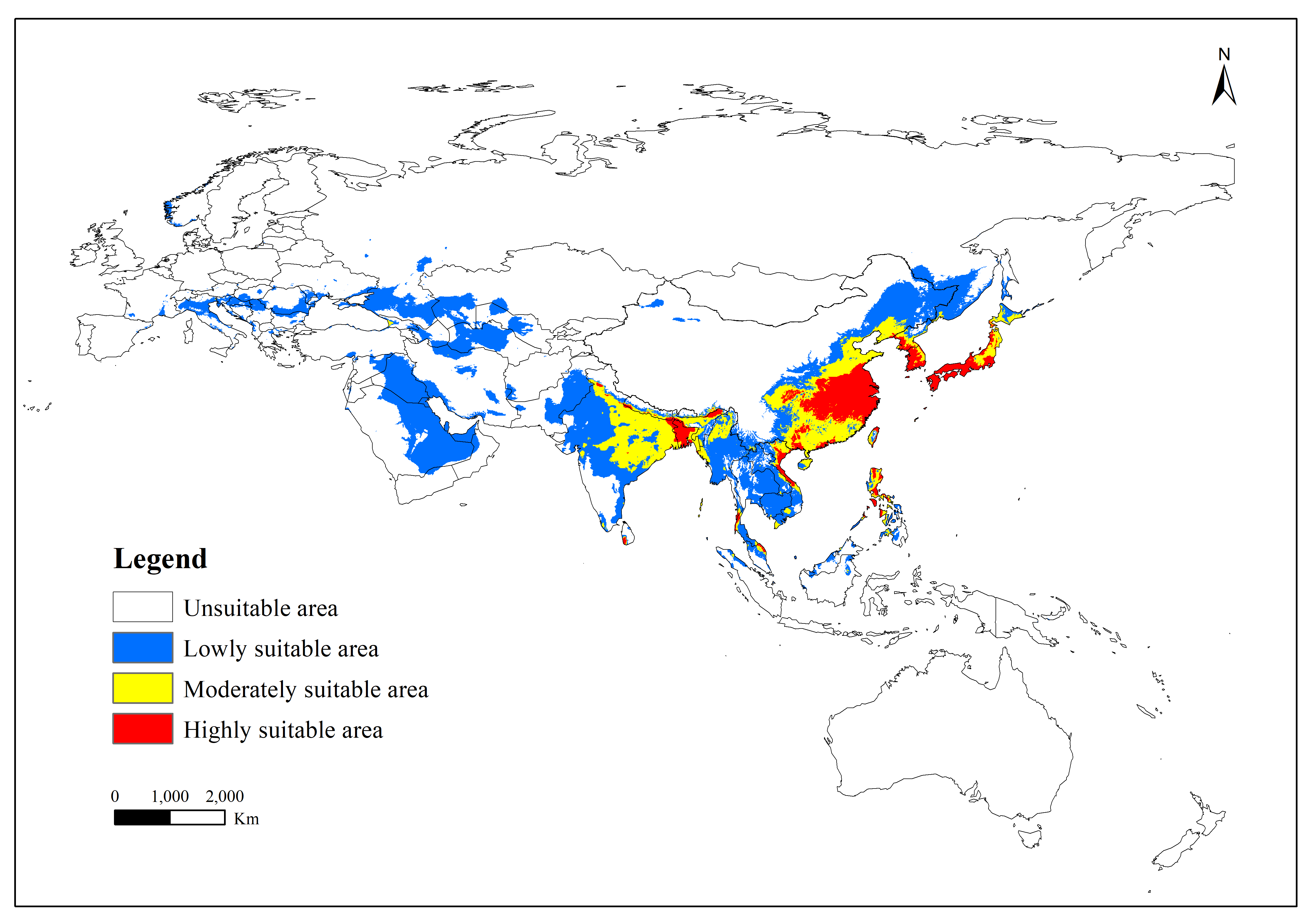

Supplement: SUPPLEMENTARY FIGURE 1 — Distribution of data points around the world. [file Data_Sheet_1.zip › Supplementary material/Supplementary files/Future distribution of Asian and neighboring countries/2081-2100 ssp245.tif]

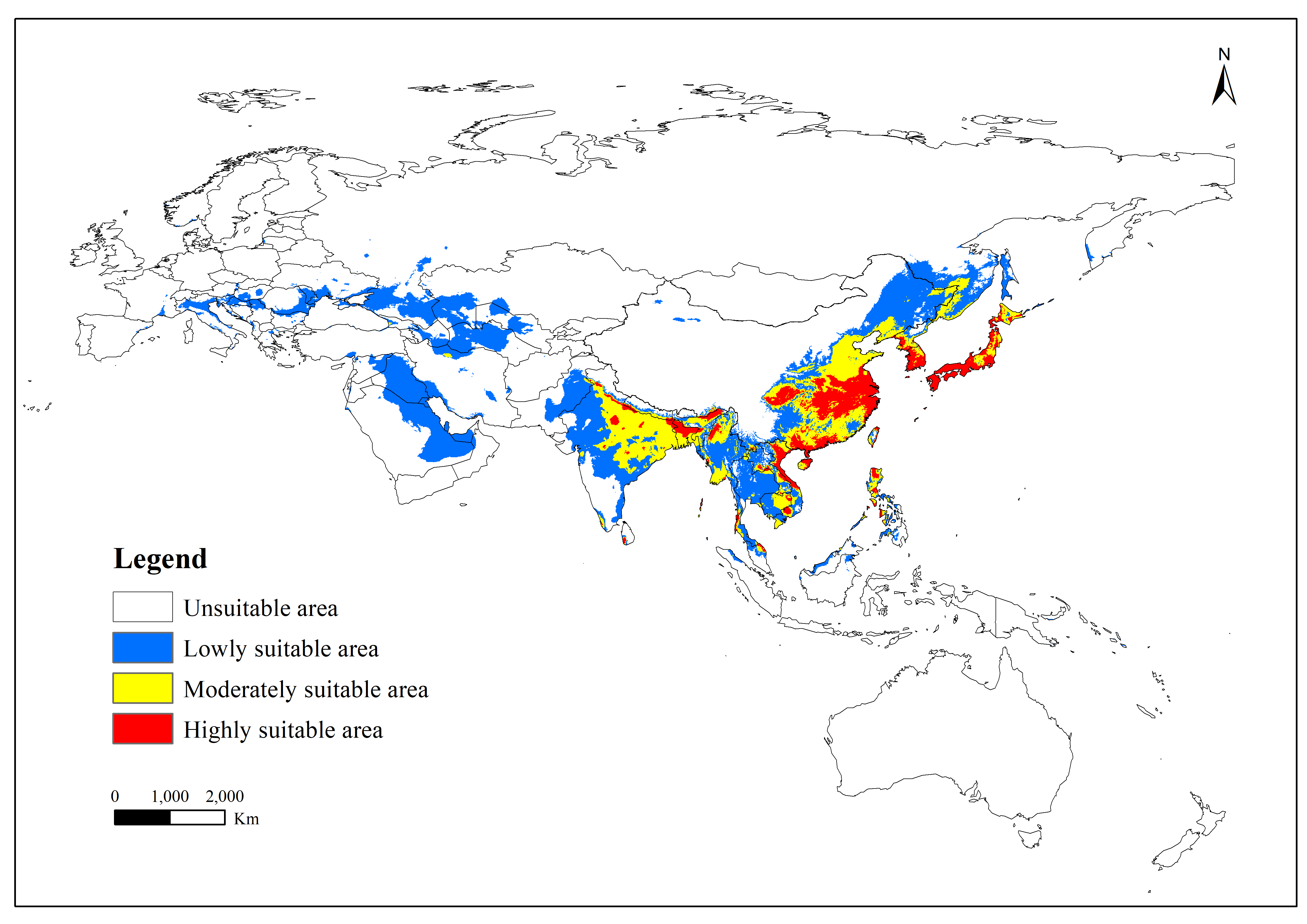

Supplement: SUPPLEMENTARY FIGURE 1 — Distribution of data points around the world. [file Data_Sheet_1.zip › Supplementary material/Supplementary files/Future distribution of Asian and neighboring countries/2081-2100 ssp370.tif]

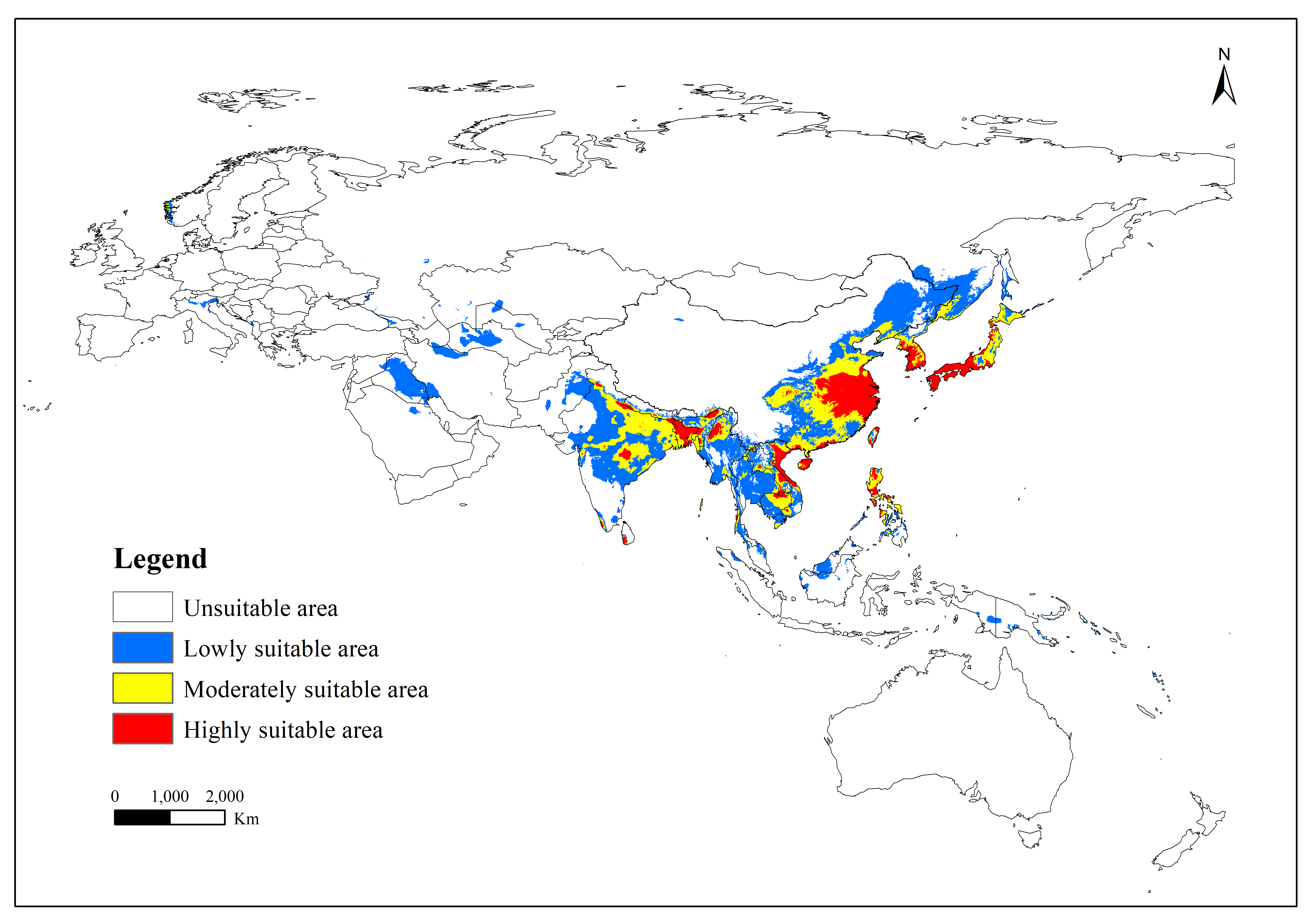

Supplement: SUPPLEMENTARY FIGURE 1 — Distribution of data points around the world. [file Data_Sheet_1.zip › Supplementary material/Supplementary files/Future distribution of Asian and neighboring countries/2081-2100 ssp585.tif]
